# Data quality self-assessment of child health and sexual reproductive health indicators in Botswana, 2016-2017

**DOI:** 10.1371/journal.pone.0220313

**Published:** 2019-08-13

**Authors:** Lebapotswe B. Tlale, Barnabas Morake, Onalethata Lesetedi, Lucy Maribe, Mabole Masweu, Cheikh Faye, Gershim Asiki

**Affiliations:** 1 Ministry of Health and Wellness, Gaborone, Botswana; 2 University of Botswana, Gaborone, Botswana; 3 World Health Organisation, Botswana Office, Gaborone, Botswana; 4 African Population and Health Research Center, Nairobi, Kenya; 5 Department of Women’s and Children’s Health, Karolinska Institute, Stockholm, Sweden; Yale University Yale School of Public Health, UNITED STATES

## Abstract

There is no published data on quality of administrative data for various health indicators in Botswana, yet such data are used for policy making and future planning. This article reports on quality of data on child health and sexual and reproductive health (SRH) indicators in Botswana. The main objective of the study was to assess the quality of administrative data from Expanded Immunization Program (EPI) and condom use, Depo-Provera uptake and domiciliary care attendance in Botswana. This was a retrospective study entailing a review of data retrieved from district health records and District Health Information System (DHIS). A total of 30 clinics and health posts were randomly selected from two cities, a town and three rural villages which makes up 6 districts commonly denoted urban, semi-urban and rural respectively. Through a stratified random sampling health facilities were selected. EPI data (Penta 3- third dose of pentavalent vaccine and Measles vaccine) and SRH data (condom use, Depo-Provera uptake and Domiciliary care) were assessed for completeness, discrepancies and verification factor using WHO Routine data quality (RDQA) assessment tool. A verification score of less than 90%% was considered as underreporting while more than 110% is over reporting. However, the score which is within +-10% is acceptable, reliable and a good indicator of data quality and reporting system. About 56% (9/16) SRH indicators had a verification factor score outside the accepted range and 87% (13/15) discrepancy value outside the accepted range. For immunization, 10% (1/10) had a verification factor score outside the accepted range and 33% (3/9) had a discrepancy value outside the accepted range. The level of completeness was high for both Penta3 and Measles coverage and it was lowest for condom. Our findings highlight a poorer data quality for SRH indicators compared to child health indicators. A comprehensive program review drawing lessons from the child health indicators is required to improve the quality of administrative data in Botswana.

## Introduction

The use of administrative data for effective planning, monitoring and supervision of health care programs will largely depend on the quality of data from health facilities. However, most low and middle income countries (LMICs) have weak systems for maintaining and reporting health facility data and end up with data that are incomplete, inaccurate, and untimely [1–4]. Thus, it is important for countries to regularly evaluate the quality of their routine administrative data in order to use such data with confidence for planning and monitoring health services.

Maternal and child health indicators are commonly used to monitor the success of health programmes at both national and subnational levels. For example, the data on immunization coverage is important for monitoring the performance of immunization services, to guide strategies for control. Furthermore for monitoring elimination and eradication of vaccine preventable diseases, identify gaps in the immunization system and assess the need to introduce new vaccines [5–10]. Similarly quality data on the uptake of family planning is needed to address the high maternal mortality occurring in LMICs [11]. Reproductive risks can be reduced substantially by preventing unwanted pregnancies through good family planning programs [11, 12].

Botswana has consistently conducted national population census every 10 years since 1971, and several Demographic surveys between censuses. These have certainly provided useful data such as infant and under 5 mortality, albeit not as detailed as the Multiple indicator Cluster Survey (MICS) and Demographic Health Survey (DHS). However, Botswana depends on program administrative data for policy making and future planning because alternative data sources such as national population based surveys are scanty or outdated. Botswana conducted only one demographic health survey (DHS) since 1988 and no multiple indicator cluster survey (MICS) has been conducted. In contrast, many countries in southern Africa conducted between three to eight national population based surveys each in the same period [13]. If accurately and timely collected, administrative data would be an important alternative to population based surveys for a number of key indicators since they provide more frequent assessments and also provide subnational assessments up to the level of smaller communities served by health facilities. National population based surveys are usually not adequately powered for local context and are collected less frequently with a data gap of at least 5–10 years in most cases.

No formal administrative data quality assessments have been conducted in Botswana. This hampers the ability to track progress in achieving sustainable development goals (SDGs) with confidence. For example the SDG 3 target for Botswana is 70 per 100 000 live births for the maternal mortality ratio and 25 per 1000 live births for the under-five mortality rate; but without reliable data it will be difficult to track progress in achieving these targets [14, 15]. In this paper, we report an assessment of the quality of administrative data for child health and sexual and reproductive health indicators (SRH) in Botswana. This is to explore how accurate and appropriate these data are for informing policy and tracking progress against the SDG targets. Through this, we contribute to the development of appropriate strategies for improving the quality of SRH and child health indicators generated through administrative data.

## Materials and methods

### Study design

This was a retrospective study involving the review of data obtained from district health records (paper based data collection tool) and District Health Information System 2 (DHIS 2). We only report on the data quality, coverage of indicators and comparison between rural and urban districts. For data quality, only data from January to December 2017 were used and for coverage indicators we used two years data from January 2016 to December 2017. Two EPI indicators (third dose of pentavalent vaccine-Penta 3 and Measles 1 coverage) and three SRH indicators (Depo-Provera uptake, condom uptake and domiciliary care visits) were selected for assessment. For EPI data we assessed the summary sheets, tally sheets and data from DHIS-2, while for SRH indicators we used the registers, summary sheets and DHIS-2.

A data quality assessment tool adapted from the Routine data quality assessment manual (RDQA) was used for data collection and part of the analysis [16]. The RDQA is a flexible toolbox that focuses on verifying the quality of reported data, assessing the underlying data management and reporting systems for standard program‐level output indicators. It assists countries to determine the accuracy of reported data and the quality of the monitoring systems. The research team collected data from the facilities. The team received training on how to collect and enter data. Data was then analysed by the lead researcher.

### Study setting and population

Botswana is a landlocked country situated in the Southern African plateau with a total population projection of 2 264 993 and of these 47 545 were children under the age of one year in 2017. The estimated number of women of childbearing age (15–44 years) according to Statistics Botswana is 635 461 [17]. The ministry of Health and Wellness is central government organ with overall responsibility for health care delivery. Health services are decentralized to the district level and delivered through a hierarchical network of health facilities, ranging from referral hospitals to district and primary hospitals, clinics and health posts. As a result of investment in health services, 84% of population live within 5 km radius to a health facility [18].

Public health services are provided at a minimal charge. Some of the services are free. Service delivery is through 674 health facilities and 1000 mobile stops. All health services including immunizations and sexual and reproductive health programs are provided at no direct cost to the clients. At facility level (mobile stops, health posts and clinics) data collection is paper based and aggregated reports are sent to the district on monthly basis. Data at facility level is collected using tally sheets and the district level aggregates the data into a summary sheet and enters the data into the DHIS. Once data are in the DHIS, access can be achieved at the national level for analysis. This reporting system applies both to public and private facilities.

### Data sampling and sample size

EPI INFO 7 was used to determine sample size and power. Out of 28 health districts in the country, a sample size of 6 districts was estimated to be representative at a 95% confidence level using the stats calculator from Epi info version 7. A two stage sampling technique was then used to select the 6 health districts; first based on location and economic status (2 urban, 2 semi urban and 2 rural districts) then five facilities were selected using simple random sampling from each of the selected districts.

### Key indicators assessed

Three indicators were used for quality of data assessment are; verification/accuracy factor, discrepancy and completeness of reporting. These were automatically calculated as and when data was entered [16].

Verification factor (VF): is the key metric for assessing the quality of the reported data by comparing the reported data available at the health facility in tally sheets or registers against the same data reported at the district in a specified period of time. This illustrates extent to which data correlates across data from all levels. Data verification has acceptable ranges between 90 and 110 percent [16]. A percent lower than 90% denotes under-reporting [16], meaning that more information was retrieved at the health facility than was reported at the district whilst a score above 110% shows over-reporting meaning that not all reported information at the district could be verified at the health facility source documents.

Discrepancy percentage: this measures the magnitude of the difference between data sources and interpreted as any figure of + or– 10% is regarded as close enough to each other. The larger the discrepancy the lower the quality of data.

The completeness of the data: this indicates if all the variables have been completely filled [16].

The coverage indicators used were; Penta 3 coverage: the third dose of pentavalent vaccine, which is a proxy for completion of the vaccination series and the ability of the health system to reach children multiple times with an essential service. This is reported monthly as a proportion. The monthly formula for Penta 3 coverage is the number of children who received penta 3 vaccine for that month divided by under 1 population divided by 12 times 100.

Measles routine immunization coverage: serves as a proxy indicator for access to basic health services for children under five years of age. This is reported monthly as a proportion. Measles routine immunization coverage is calculated as the number of children vaccinated divided by population under one (for a specified year) multiplied by 100. It serves as a proxy indicator for access to basic health services for children under five years of age.

Depo Provera coverage: is the number of women of childbearing age receiving the Depo Provera injectable contraception. This is recorded as an absolute number and is reported monthly.

Condom use: refers to the distribution of male condoms to adults accessing health services. This is reported monthly in absolute numbers. Condom use is calculated as the number of male condoms distributed in a specified year.

Domiciliary Care is the number of home visits provided by health workers to a woman who delivered at their facilities. It is recorded monthly as an absolute number. Domiciliary Care is calculated as a count of home visits provided by health workers to a woman who delivered at their facilities.

### Data analysis

Data were entered into Microsoft excel 2013 and RDQA tool. Data were analysed using RDQA tool and STATA version 12 (College Station, TX). The data quality assessment tool is divided into five components namely; health facility, month, completeness, data collection form health facility, district level and health information system [16]. The data collectors retrieved information from child health and SRH sources of data collection tools (tally and summary sheet and DHIS 2) and re-entered the numbers in the form per antigen. This process compares recounted data with the original data. Following this procedure, the form simultaneously auto calculates: Distribution of coverage indicators by geographical location and trends overtime were computed using STATA and Excel.

### Ethical considerations

Approval for the research was given by ethics committees and the Health Research and Research Committee (HRDC) at the Ministry of Health and Wellness. An expedited review was granted because the study did not pose any risk or harm since the researchers only accessed aggregated data with no individualised and personal identifiers. There was no data obtained directly from patients or patients’ records hence consent was not necessary.

## Results

### Characteristics of health facilities in the study

A total of 30 health facilities were selected from the 6 districts. Of these, six offered domiciliary care, 27 family planning (Depo-Provera and Condoms) and 27 offered Immunizations (Penta 3 and Measles). Two health facilities had been closed and one of these was a private facility which did not offer family planning or vaccinations. The two facilities were from the semi- urban stratum.

### Reporting consistency from health facilities to national level

The verification factor and discrepancies for all the 5 indicators are shown in Table 1 and Table 2 respectively. About 56% (9/16) SRH indicators had a verification factor score outside the accepted range and 87% (13/15) had a discrepancy value outside the accepted range. Five were due to under-reporting and 4 to over-reporting. For EPI about 10% (1/10) had a verification factor score outside the accepted range and 33% (3/9) had a discrepancy value outside the accepted range. Two (2) were due to under-reporting and one (1) due to over-reporting. Furthermore, the immunisation coverage decreased both for measles and penta3 for the year 2017, from 95% to 80% and 89% to 79% for measles and Penta 3 respectively.

**Table 1:** Verification factor and discrepancy values in percentages for EPI indicators for selected districts in Botswana, January–December 2017.

District	Penta 3		Measles 1	
	Verification Factor	Discrepancy	Verification Factor	Discrepancy
Francistown	96	4	104	4
Gaborone	110	25^b^	101	2
Palapye	n/a	n/a	89	16^b^
Kweneng east	n/a	n/a	93	9
Kweneng west	400^a^	300^b^	103	9
Mabutsane	100	Na	102	2

^a^ Values outside the accepted value range of 90% to 110%

^b^values outside Accepted value is + or– 10%

**Table 2:** Verification factor and discrepancy values in percentages for SRH indicators for selected districts in Botswana, January–December 2017.

District	Domiciliary		Depo Provera		Condom use	
	Verification Factor	Discrepancy	Verification Factor	Discrepancy	Verification Factor	Discrepancy
Francistown	96	4	51^a^	49^b^	27^a^	73^b^
Gaborone	110	25^b^	101	15^b^	86^a^	43^b^
Palapye	n/a	n/a	90	24^b^	130^a^	106^b^
Kweneng east	n/a	n/a	85^a^	57^b^	88^a^	50^b^
Kweneng west	400^a^	300^b^	125^a^	38^b^	136^a^	48^b^
Mabutsane	100	n/a	101	11^b^	102	10

^a^Values outside the accepted value range of 90% to 110%

^b^values outside Accepted value is + or– 10%

Verification factor for measles 1 is all within the acceptable limits while for Penta-3. Kweneng west, had VF outside the acceptable limit. Palapye and Kweneng east had no data for Penta-3. Discrepancies in data were observed in Gaborone and Kweneng west for penta3 and in Palapye for measles 1 vaccination.

Domiciliary care in Kweneng west (Rural District) and Mabutsane (Rural Centre) recorded the highest verification factor. Francistown recorded the lowest verification factor. Regarding condom use, besides Mabutsane all districts had both VF and discrepancy outside the acceptable range.

### Completeness of reporting indicators

**Fig 1:**
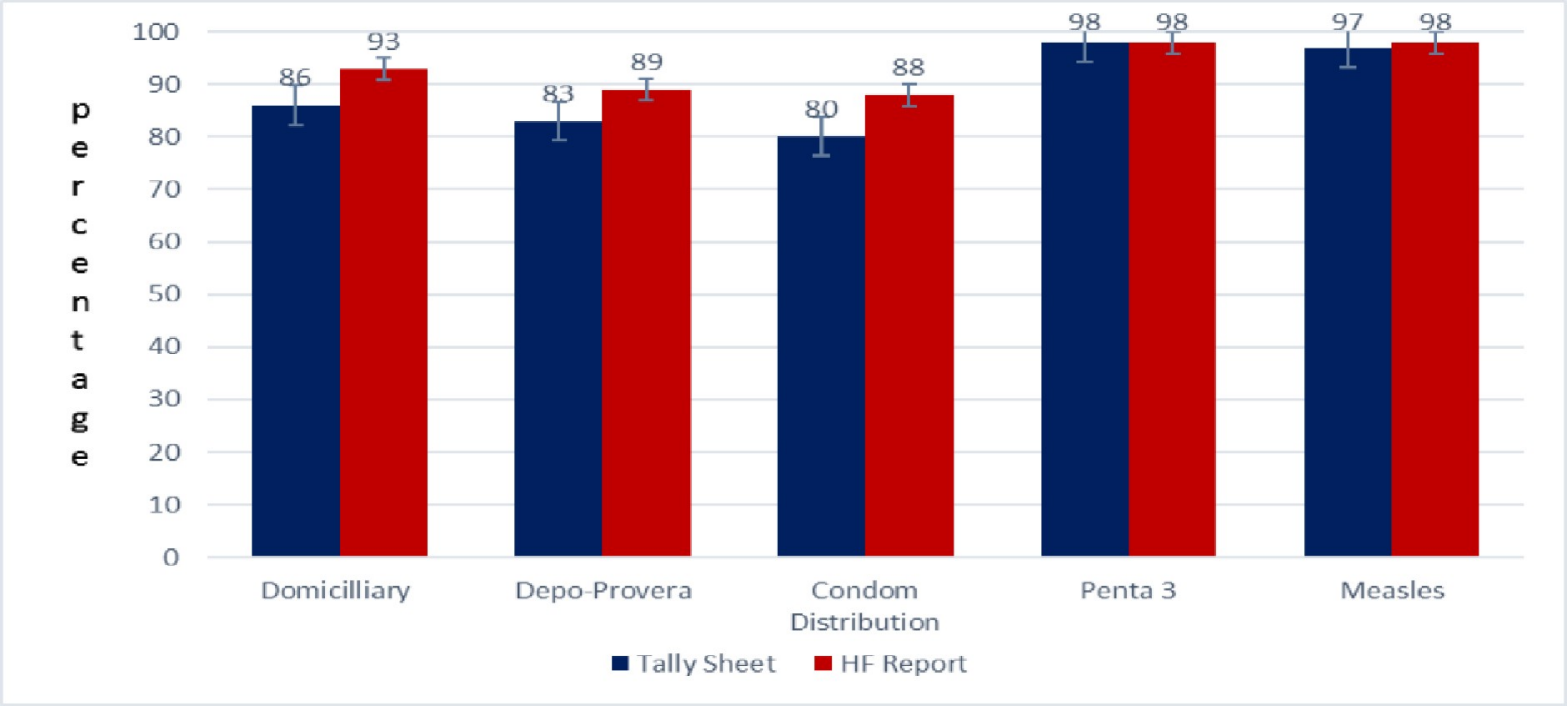
The level of completeness of reporting for SRH indicators and EPI indicators in Botswana, 2016–2017. The blue bars show data from tally sheets and the red bars show the health facility data from DHIS-2.

Completeness of reporting was high for all indicators (more than 80%) but the level of completeness was higher for both EPI indicators and did not vary between tally sheets and health facility reporting. For SRH indicators tally sheet reporting was slightly lower than health facility reporting. Completeness of reporting was lowest for condom use both in the tally sheet and facility report, followed by Depo Provera. (Fig 1).

### Indicator coverage by geographical location

#### Coverage distribution by geographical location

**Fig 2:**
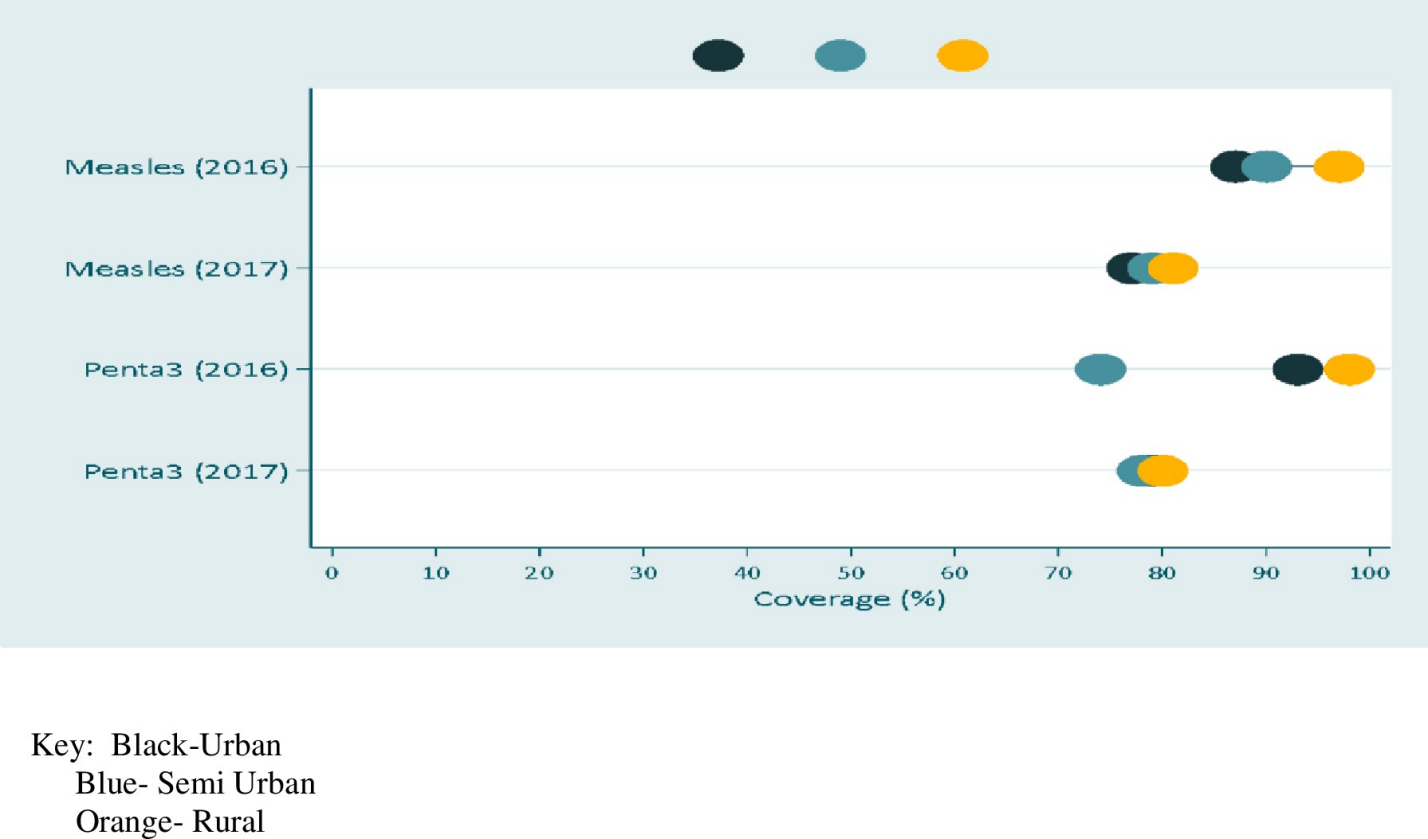
Coverage of measles and Penta 3 vaccinations by location of the districts. The black circles represents coverage of measles of either Penta 3 or measles coverage in urban areas, the blue circles represents the coverage in semi urban areas and the orange represent coverage in the rural areas.

There were few disparities between the districts according to their location especially in 2016. For Penta 3, semi-urban areas had the lowest coverage followed by rural and then urban in 2016. While for measles vaccination coverage the rural and semi-urban districts had relatively similar coverages but lower than the urban areas in 2016. In 2017, coverage did not vary by location of the district for both Penta3 and measles vaccinations (Fig 2).

**Fig 3:**
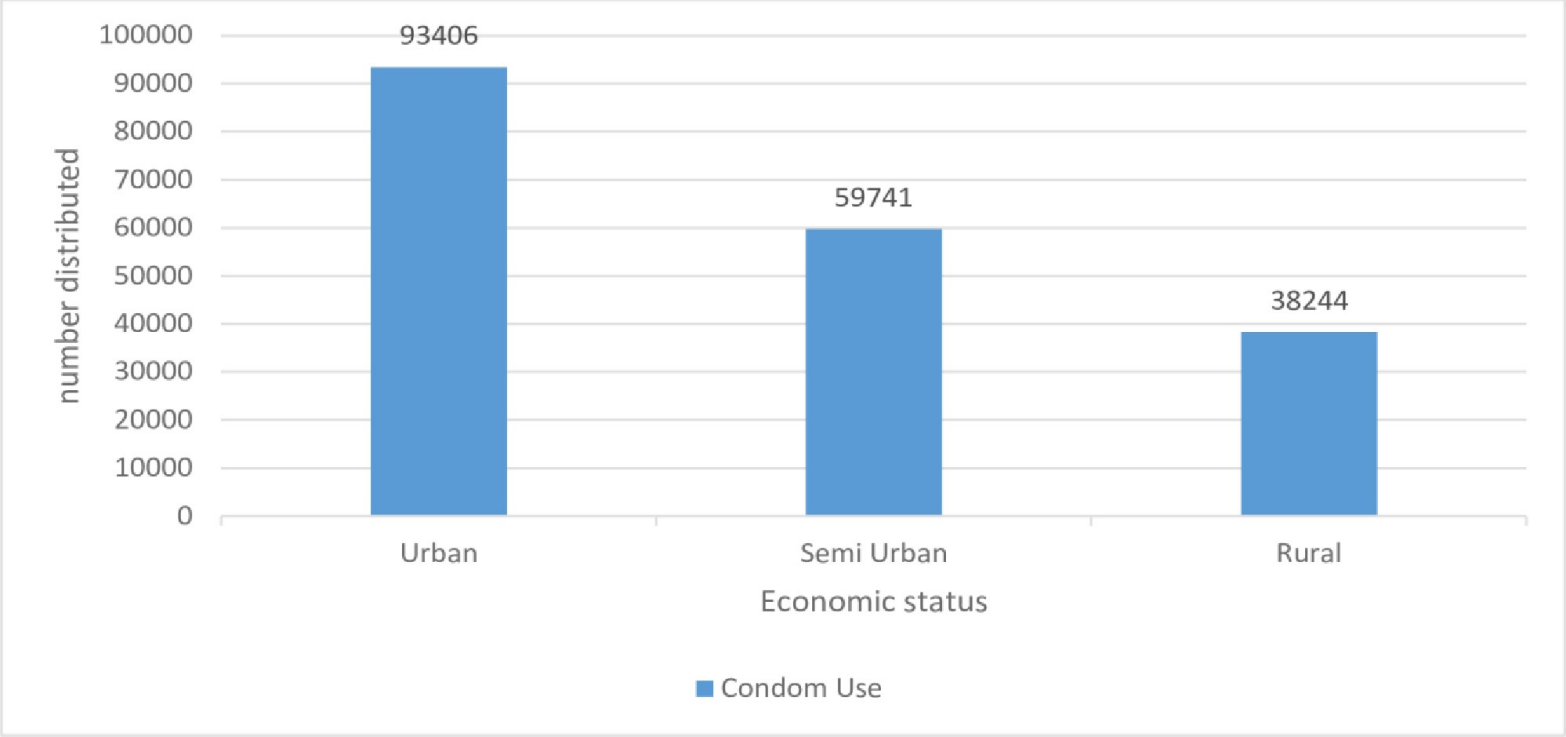
Condom distribution by district location, 2017.

**Fig 4:**
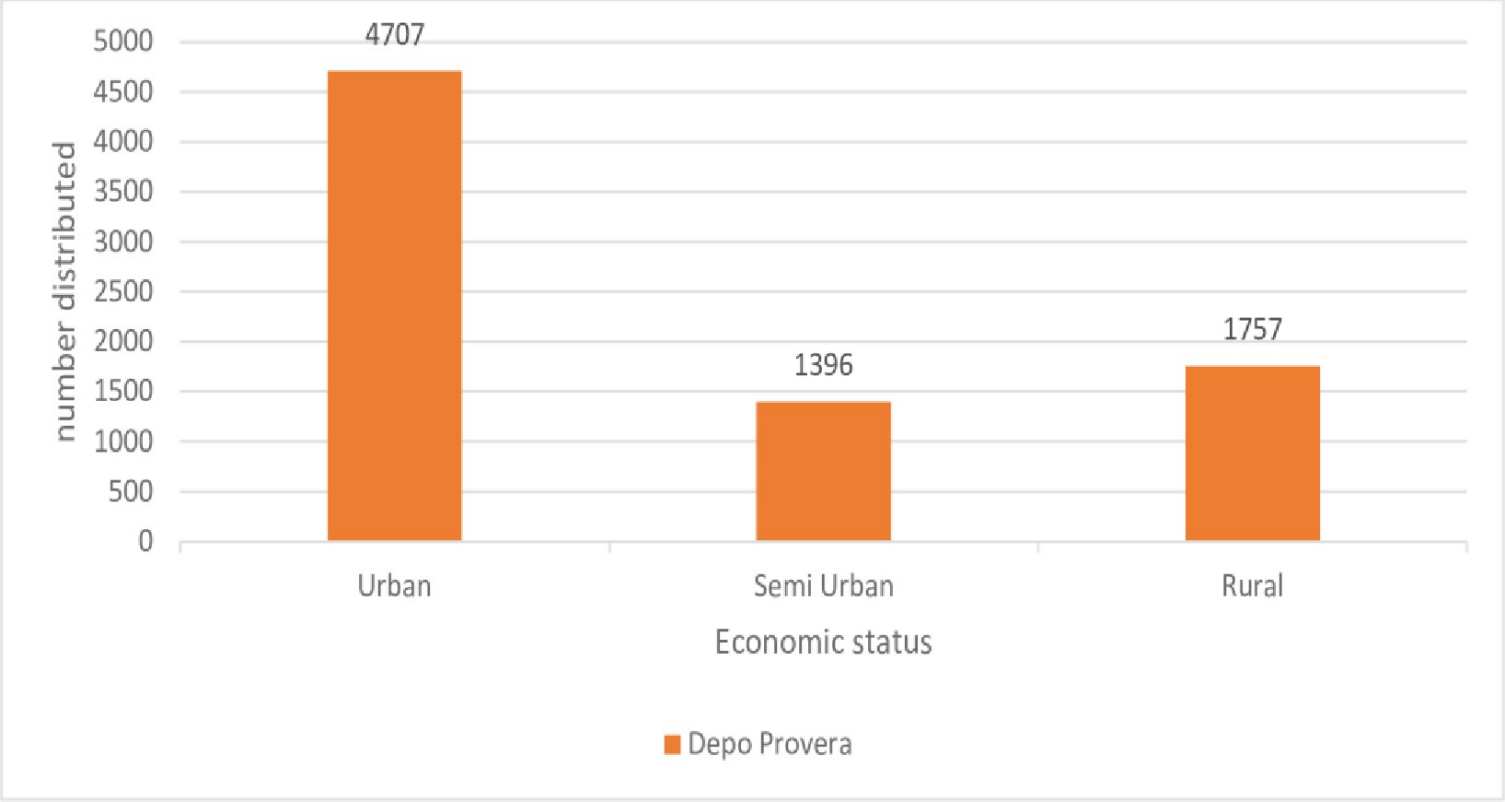
Depo-Provera distribution by district location, 2017.

**Fig 5:**
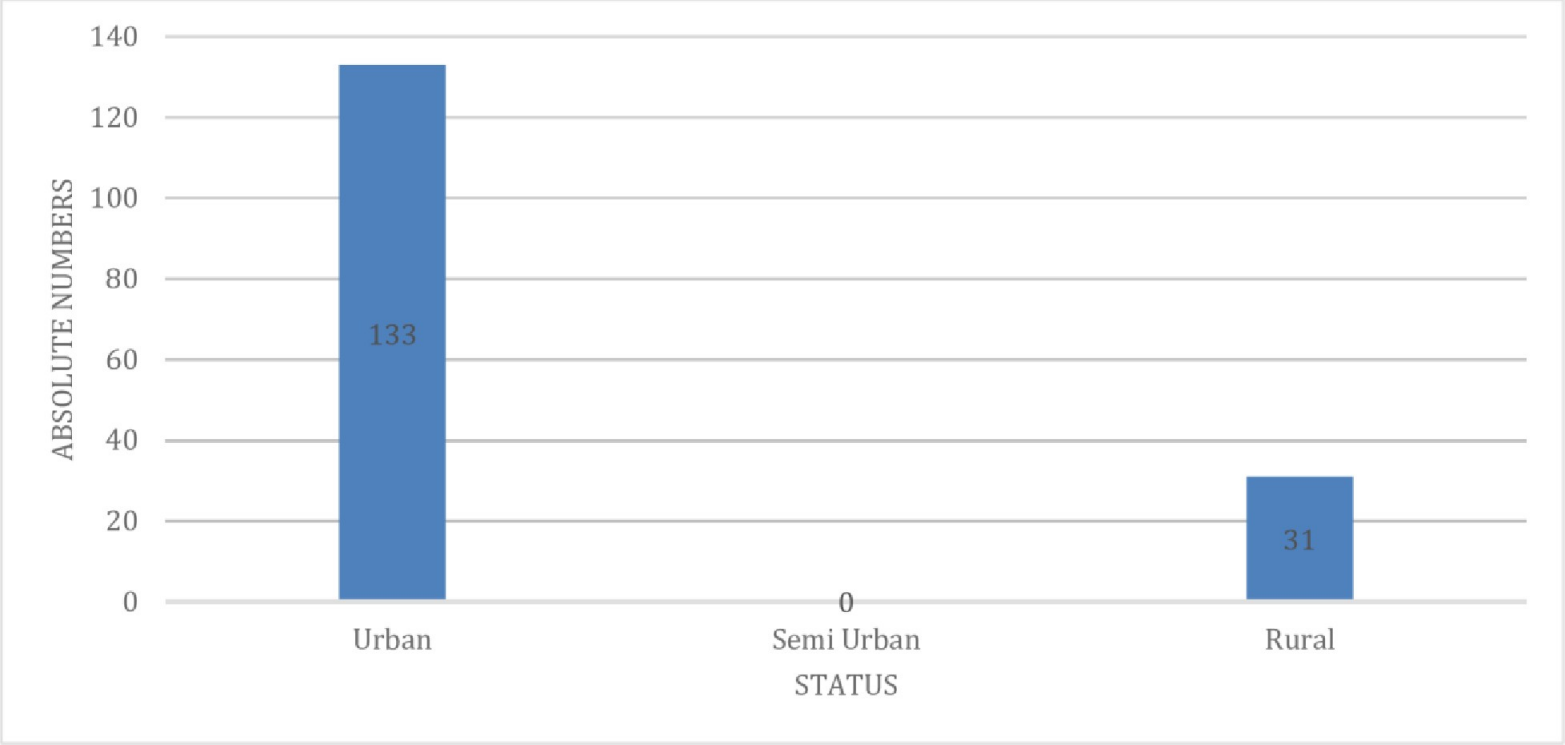
Domiciliary care by district location.

Condom distribution was very high in the urban area and very low in the rural areas. Depo-Provera distribution was high in the urban area and lowest in the Semi urban. Domiciliary care was higher in urban districts approximately three times higher in the urban districts compared to rural districts (133 versus 31) (Figs 3, 4 and 5).

#### Trends in the coverage distribution between 2016 and 2017 by district

**Fig 6:**
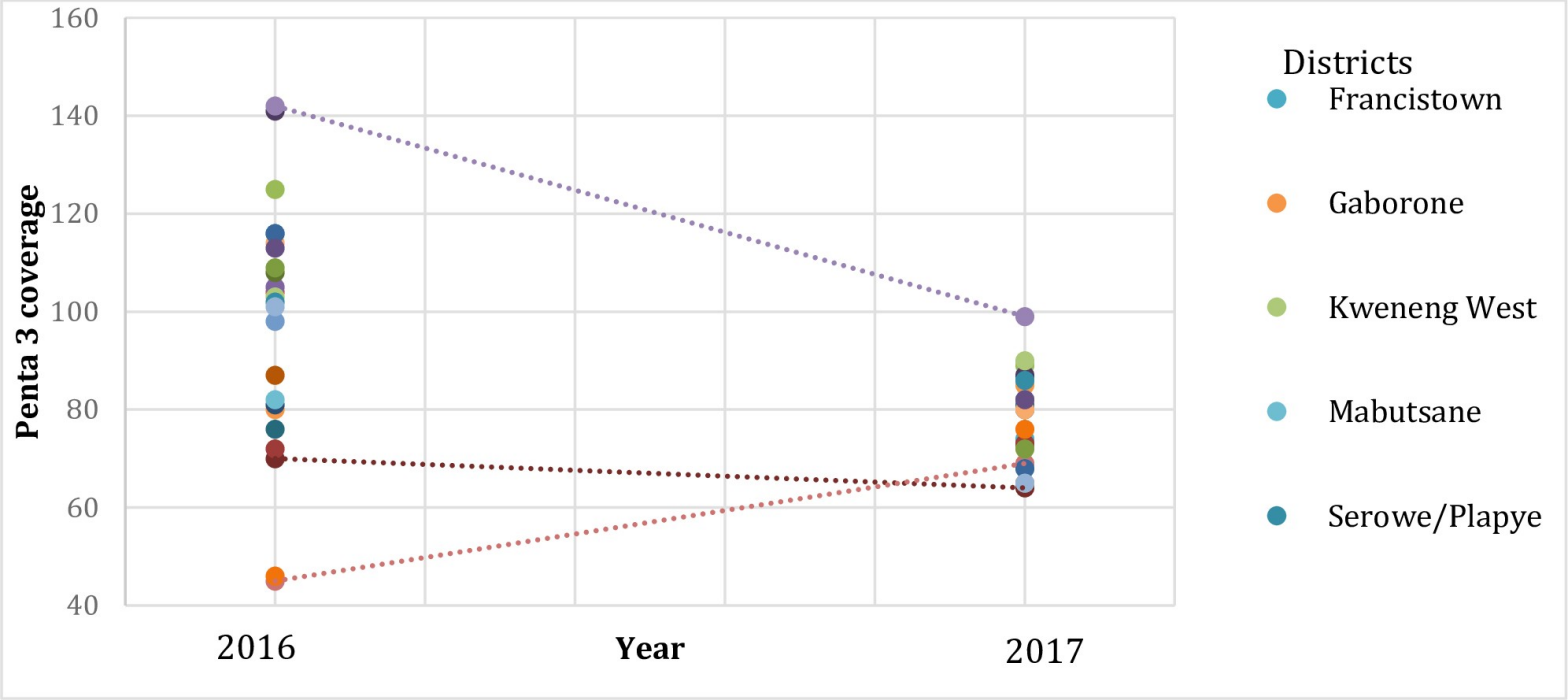
Comparison of coverage for Penta3 by district, 2016–2017. The different dots represents coverage of Penta 3 by health districts in 2016 and in 2017.

The trend tended to vary from one district to another. For example, For Gaborone, there was a substantial increase in coverage from 2016 to 2017 while some districts had a drop in coverage and others had did not show any change in coverage (Fig 6).

## Discussion

The most prominent feature from our assessment is that the quality of child health indicators is better than that of sexual and reproductive health. About half of the SRH indicators had a verification factor score (VF) outside the accepted range and discrepancy value outside the accepted range. This mainly resulted from under reporting as evidenced by VF outside of range for most indicators.

For EPI, one in ten of indicators had a verification factor score outside the accepted range and one in 3 of indicators had a discrepancy value outside the accepted range. The level of completeness for all the 5 indicators is acceptable as is above 80% for all indicators. Data quality remains a challenge in developing countries and this is also evident in this paper [19]. A study comparing routine and demographic health survey data of 45 countries using DTP 3 (now Penta 3 in most countries) showed that there is over-reporting of routine administrative data and the size of the difference increases with the rate of reported coverage of DTP3 [20]. This difference could be attributed to the transition from DPT to DPT-Hib-HepB (Pentavalent) vaccine and the introduction of measles second dose into Botswana routine immunization. The Botswana Comprehensive Multi Year (cMYP) plan found out that there was a delay in updating reporting tools which could be attributed to the differences [21]. There is urgent need for independent and contestable monitoring of health indicators in an era of target-oriented and disbursement of funds global initiatives based on performance [22]. Poor data quality (DQ) can have substantial social and economic impacts [23].

The reasons for the wide variation in the quality of data for sexual reproductive health and child health indicators could not be established within the scope of this work. One plausible explanation is the differences in the data collection tools. In Botswana data is mainly collected by nurses at facility or lower level of care. Programs collect their own data (there is no centralised monitoring and evaluation system). EPI uses tally and summary sheet which have few indicators as compared to SRH registers and summary sheet which requires a lot of information to be filled.

The difference in quality of data directly reflected on the achievement of millennium development goals. Botswana was able achieve Millennium development goal 4 of Child mortality but failed to reach the Millennium development goal number 5 (maternal mortality) [18]. Currently the 2016 maternal mortality ratio is 156.6 per 100 000 live births and it may be difficult to achieve the SDG target if quality for SRH is not improved [24]. There is need to find strategies to improve the quality for SRH indicators. Surprisingly, there was no substantial difference in the level of completeness of reporting between SRH and child health indictors. This attests to the fact that health facilities are compliant with reporting both indicators but the challenge may be with the type of tools and capacity of health workers collecting the data used for SRH indicators.

The two programs are run as vertical program, thus reporting and working in silos. A cross sectional review of Benin routine health information found out that vertical programs’ reporting systems and monitoring tools provide insufficient data to the health system hence poor quality. Furthermore, the findings suggest that this leads to delayed decision making and often faulted for poor reliability and accuracy [19]. They also stated that data quality is insufficient specifically in routine health information for developing countries; with sub Saharan African more challenged [19]. Secondly, poor data quality is attributed to paper based reporting and sub-optimal utilization of computerization [25]. Poor data quality can result in loss of money and life because inaccurate and insufficient data will be utilized for planning [26]. Therefore, evidence is unanimous that integrated health care information systems for data quality improvement and decision support need to prioritized as a matter of urgency [26].

Immunisation coverage in urban areas was higher than in the rural areas for the year 2016, however the coverage was very similar in urban and rural areas for the year 2017. In other words, no inequalities between the districts existed. The higher coverage in the urban areas in 2017 may be explained by the ease of access to information and immunisation services [27]. Lack of knowledge or information has been implicated as one of the obstacles to receiving immunizations [21, 27]. Lack of outreach and community mobilization have been documented as the contributing factors to decline in immunizations coverage in Botswana in rural areas [16, 21]. Under reporting at subnational level is also a major challenge to the low immunization coverage rates [21]. The major difference between immunization coverage’s in 2016 and 2017 can be attributed to data quality issues arising from unrealistic denominator population projections. Before 2017, the denominator for coverage has always been a challenge because of the difference in administrative and health districts [21, 27]. This might also be the reason for the difference in equality between the urban, semi urban and rural areas in 2016 and 2017. Accessibility and availability of resources in rural versus urban areas contributes to inequalities to child or maternal care in developing countries [28].

Botswana like other Sub-Saharan countries has been hard hit by the HIV/AIDS epidemic. This might have led to the promotion of barrier methods for family planning as the behavioural intervention to reducing HIV transmission as the method of choice, hence higher number of condom users compared to Depo-Provera users. The higher use of these methods in urban settings might be due to a higher population compared to semi urban or rural areas as absolute numbers are used instead of proportions for this indicator. Absolute numbers do not communicate well whether the intended target or goal is been reached compared with proportions, rate and ratios. The reason for choosing these condoms and Depo- Provera for assessment is that there has been a shift in the past decade away from sterilization towards injectable drugs and barrier methods [29].

The reason for using absolute numbers is that there are various family planning methods that an individual might choose at a specific period in time and it is usually difficult to come up with a denominator. Domiciliary care program has not been given much attention. Studies have shown that antenatal and postnatal care can be considerably improved through implementing interventions at family and community levels, including health education to improve domiciliary neonatal care practices and health seeking behaviour for neonatal illness [30]. WHO recommends that the mother and baby be visited at home by a trained health worker preferably within the first week after birth [31]. These have been shown to identify critical signs and symptoms to prevent maternal and child mortality as well as promoting breastfeeding and address any difficulties with attachment and positioning [31]. Despite this knowledge, domiciliary care continues to be inadequate in Botswana as it is evident from this study. This is an area of maternal and child health that requires a lot of attention.

The potential limitation of this review is the possibility of missing and incomplete data. Data collection is also subject to seasonal variation in immunization and family planning uptake. This required data to be computed by month for all the districts combined.

## Conclusion

In conclusion, we successfully assessed data quality of administrative data for child health and sexual and reproductive health indicators in Botswana. No previous literature has been published that answers the research question posed in our study. The findings of our study show that data quality is a challenge in both the child and SRH indicators.

Our recommendations are that comprehensive program review is required which mainly focuses on the monitoring and evaluation of both child and SRH indicators. Considering that child health indicators were more accurate than SRH indicators, some lessons could be drawn from the data collection and reporting systems for child health indicators to improve the quality of SRH indicators. There is need to centralise monitoring and evaluation system for the various health indicators and regularly evaluate the quality of data. Further research is needed to establish the factors contributing to poor data quality to inform strategies that would improve data quality.

## Supporting information

S1 Fig Gaborone domiciliary data. (TIF)

S2 Fig Gaborone Depo-Provera data. (TIF)

S3 Fig Gaborone condom use data. (TIF)

S4 Fig Gaborone Penta data. (TIF)

S5 Fig Gaborone measles data. (TIF)

S6 Fig Francistown domiciliary data. (TIF)

S7 Fig Francistown Depo-Provera data. (TIF)

S8 Fig Francistown condom use data. (TIF)

S9 Fig Francistown Penta data. (TIF)

S10 Fig Francistown measles data. (TIF)

S11 Fig Palapye domiciliary data. (TIF)

S12 Fig Palapye Depo-Provera data. (TIF)

S13 Fig Palapye condom use data. (TIF)

S14 Fig Palapye Penta data. (TIF)

S15 Fig Palapye measles data. (TIF)

S16 Fig Kweneng east domiciliary data. (TIF)

S17 Fig Kweneng east Depo-Provera data. (TIF)

S18 Fig Kweneng east condom use data. (TIF)

S19 Fig Kweneng east Penta data. (TIF)

S20 Fig Kweneng east measles data. (TIF)

S21 Fig Kweneng west domiciliary data. (TIF)

S22 Fig Kweneng west Depo-Provera data. (TIF)

S23 Fig Kweneng west condom use data. (TIF)

S24 Fig Kweneng west Penta data. (TIF)

S25 Fig Kweneng west measles data. (TIF)

S26 Fig Mabutsane domiciliary data. (TIF)

S27 Fig Mabutsane Depo-Provera data. (TIF)

S28 Fig Mabutsane condom use data. (TIF)

S29 Fig Mabutsane Penta data. (TIF)

S30 Fig Mabutsane measles data. (TIF)

## References

[1] Burton A, Monasch R, Lautenbach B, Garic-Dobo M, Maryanne N, Karimov R et alWHO and UNICEF estimate of national infant immunization coverage: Methods and Processes. Bull world health organ. 2009; 87:535–541. 10.2471/BLT.08.053819 19649368 PMC2704038

[2] Munya A, Nielsen P. Reporting Practices and Data Quality in Developing Countries: An Exploratory case Study in Kenya, Journal of health Informatics in Developing Countries. 2016 Vol 10:No 1

[3] Arts D G, De Keizer N F, Scheffer G J. Defining and improving data quality in medical registries: A literature review, case study, and generic framework. Journal of the American Medical Informatics Association. 2002;9(6), 600–611 10.1197/jamia.M1087 12386111 PMC349377

[4] Kihuba E, Gathara D, Mwinga S, Mulaku M, Kosgei R, Mogoa W, et al English M. Assessing the ability of health information systems in hospitals to support evidence-informed decisions in Kenya. Global health action, 2014 7:10. 10.3402/gha.v7.24859 PMC4119289 25084834

[5] Global polio eradication initiative, Strategic Plan 2004–2008. Geneva: World Health Organisation; 2003

[6] Measles mortality reduction and regional elimination, strategic plan 2001–2005. Geneva: World Health Organisation, United nations Children’s Fund: 2001

[7] Maternal and neonatal tetanus elimination by 2005. Geneva: World Health Organisation: 2000

[8] Vandelaer J, Bilous J, Nshimirimane D. The Reaching Every District (RED) approach as a way to improve immunization performance. Bull world health organ 2007;86 10.2471/BLT.07.042127 PMC2647411 18368190

[9] Global immunization vision and strategy, 200–2015. Geneva: World Health Organisation: 2005

[10] Vaccine introduction guidelines: adding a vaccine to a national immunization programme, decision and implementation. Geneva: World Health Organisation; 2005 (WHO/IVB/05.18).

[11] Potts M. Can family planning reduce maternal mortality? J Obstet Gynaecol East Cent Africa. 1986; 5(1–2):29–35. 12281232

[12] Winikoff B, Sullivan M. Assessing the role of family planning in reducing maternal mortality. Stud Fam Plann. 1987; 18(3):128–43. 3617120

[13] https://dhsprogram.com/data/STATcompiler.cfm 13 May 2018 11:15:50 GMT

[14] You D, Hug L Ejdemyr S, Idele P, Hogan D, Mathers C, et al Global, regional, and national levels and trends in under-5 mortality between 1990 and 2015, with scenario-based projections to 2030: a systematic analysis by the UN Inter-agency Group for Child Mortality Estimation. The lancet. 2015; 386:10010. 10.1016/S0140-6736(15)00120-8 26361942

[15] Boeldosser-Boesch A, Bru M, Carvajal M, Chou D, De-Bernis L, Kogg K, et al Setting maternal mortality targets for the SDGs. The Lancet. 2017; 389: 10010 (696–697). 10.1016/S0140-6736(17)30337-9 28229871

[16] United States Agency for International Development. User Manual: Routine Data Quality Assessment. USAID 10 2015

[17] Statistics Botswana. Population and Housing Census 2011: Dissemination Seminar. Statistics Botswana 2013

[18] Republic of Botswana. Botswana: Millennium Development Goals Status Report 2015: Sustaining progress to 2015 and beyond. 2015

[19] Ahanhanzo Y G, Ouendo E M, Kpozèhouen A, Levêque A, Makoutodé M, Dramaix-Wilmet M. Data quality assessment in the routine health information system: an application of the Lot Quality Assurance Sampling in Benin. Health Policy and Planning. 2014: 30, 837–842. 10.1093/heapol/czu067 25063699

[20] Murray C, Shengelia B, Gupta N, Moussari S, Tandon A, Thieren M. Validity of reported vaccination coverage in 45 Countries. The Lancet. 2003 362:9389 10.1016/S0140-6736(03)14411-X 14522532

[21] Lim S, Stein D, Charrow A, Murray C. Tracking progress towards universal childhood immunisation and the impact of global initiatives: a systematic analysis of three-dose diphtheria, tetanus, and pertussis immunisation coverage. The Lancet. 2008 362:9655 10.1016/S0140-6736(08)61869-3 19070738

[22] Ministry of health, Botswana. The Botswana Multi-Year Immunization Plan 2018–2022. Gaborone

[23] Wang R, Strong D. Beyond Accuracy: What Data Quality Means to Data Consumers, Journal of Management Information Systems. 1996 12:4, 5–33.

[24] Statistics Botswana. Botswana—Maternal Mortality Ratio. 2016. http://www.statsbots.org.bw

[25] Hotchkiss D. Aqil A. Lippeveld T. Mukooyo E. Evaluation of the Performance of Routine Information System Management (PRISM) framework: evidence from Uganda. BMC Health Services Research. 2010: 10, 188 10.1186/1472-6963-10-188 20598151 PMC2904760

[26] Lin B. Chan H. Managing data quality in the health care industry: Some critical issues. Journal of International Information Management. 2000: 9(1), 33–43.

[27] Ministry of health, Botswana. Botswana Measles Rubella and Deworming: Post Campaign Coverage Survey. 2016. Gaborone

[28] Houwelling T, Ronsmans C, Campbell O Kunst A. Huge poor-rich inequalities in maternity care: an international comparative study of maternity and child care in developing countries. Bulletin of the world health organisation. 2007;85:745–754 10.2471/BLT.06.038588 PMC2636501 18038055

[29] Jacqueline E, Darroch D Singh S. Trends in contraception need and use in developing countries in 2003, 2008 and 2012: an analysis of national surveys. The lance. 2013;381;1756–1762 10.1016/S0140-6736(13)60597-8 23683642

[30] Darmstadt G, Syed U, Patel Z, Kabir N. Review of domiciliary newborn-care practices in Bangladesh.PMC. 2006 24(4): 380–393 PMC3001142 17591335

[31] World Health Organisation. Counselling for Maternal and Newborn health care: A handbook for building skills. Geneva: World Health Organisation 2013 11 26158182

