# Supplementary figures and images for: Data quality self-assessment of child health and sexual reproductive health indicators in Botswana, 2016-2017
Source: PLoS One. 2019 Aug 13;14(8):e0220313. doi: 10.1371/journal.pone.0220313 (PMC6692026; doi:10.1371/journal.pone.0220313)

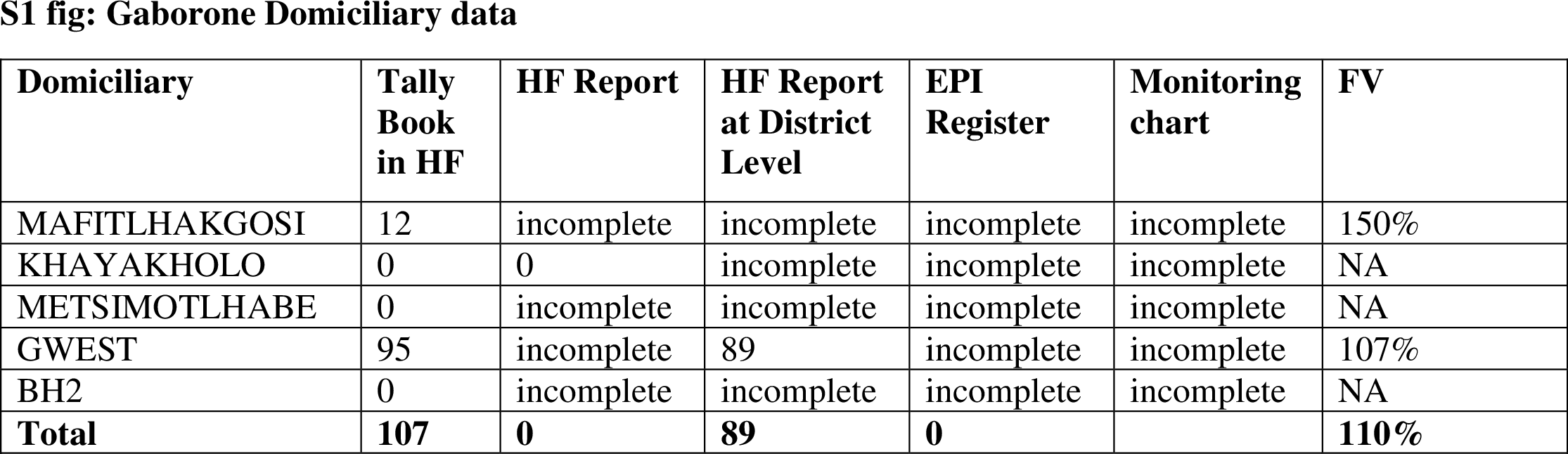

Supplement: S1 Fig — (TIF) [file pone.0220313.s001.tif]

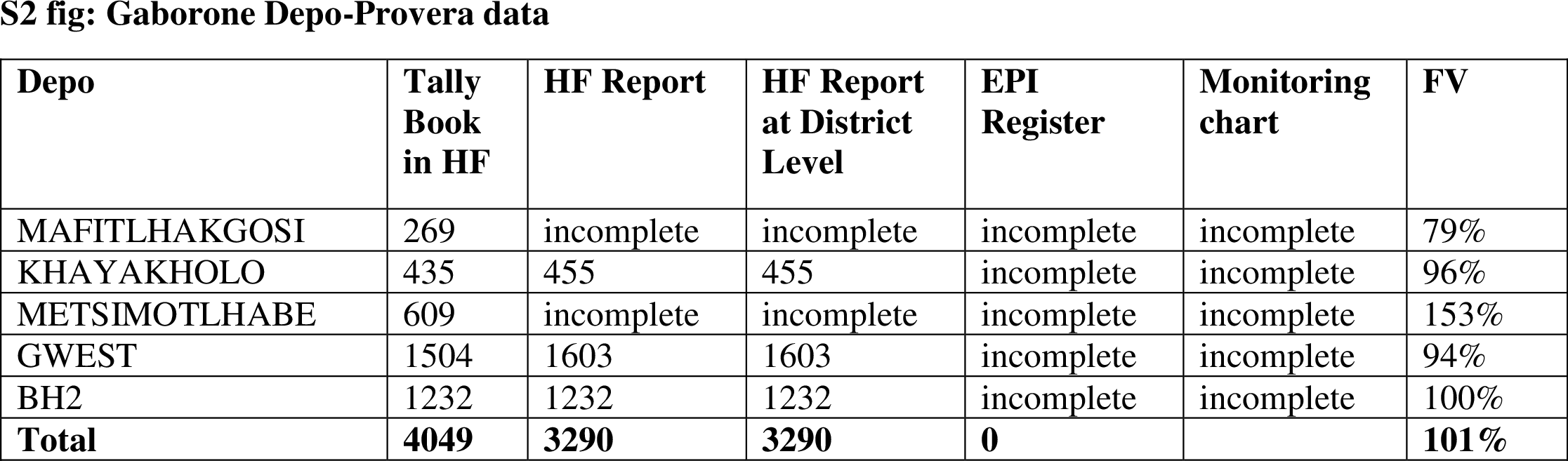

Supplement: S2 Fig — (TIF) [file pone.0220313.s002.tif]

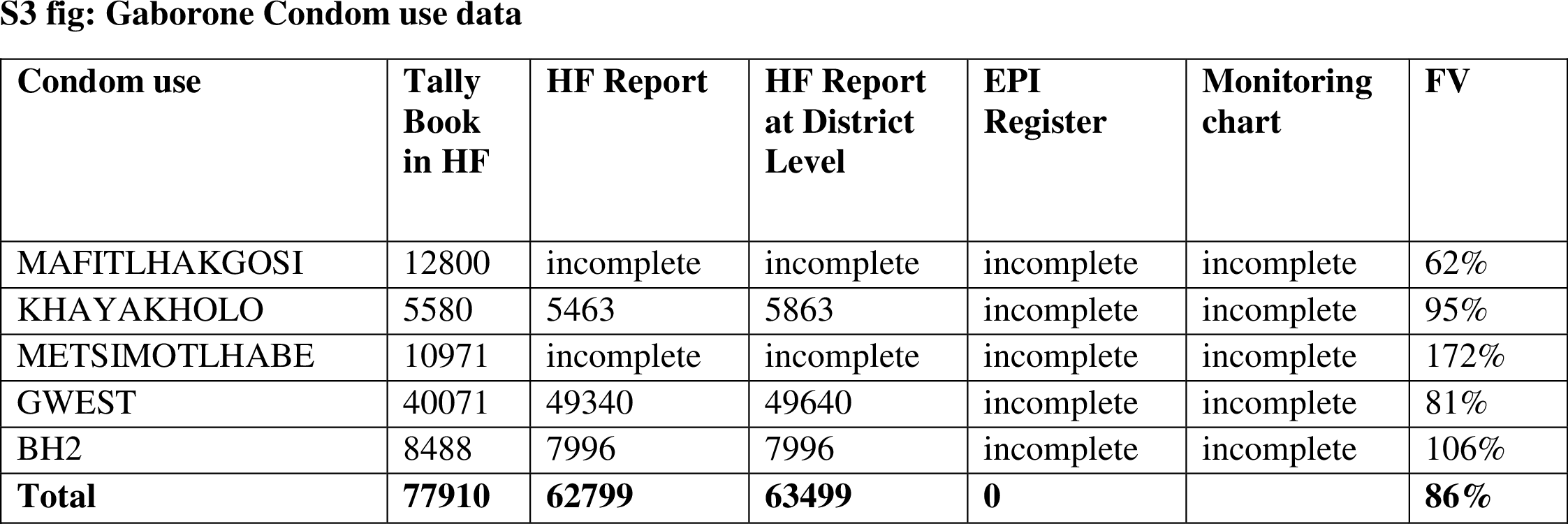

Supplement: S3 Fig — (TIF) [file pone.0220313.s003.tif]

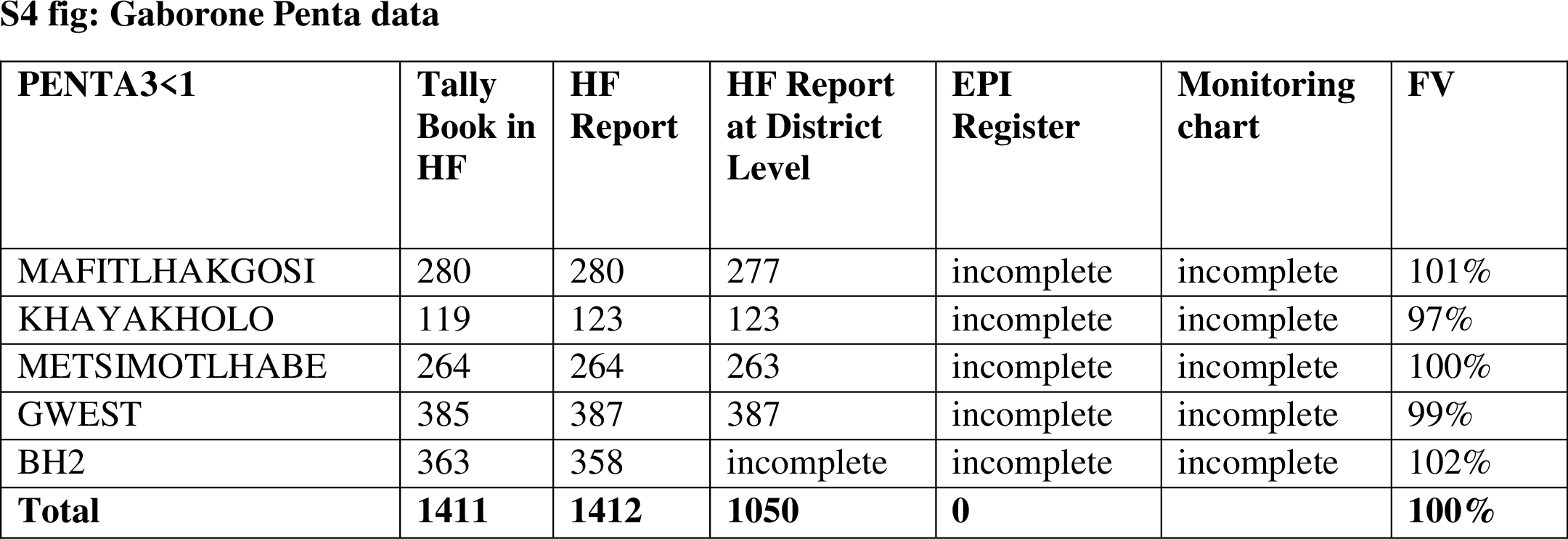

Supplement: S4 Fig — (TIF) [file pone.0220313.s004.tif]

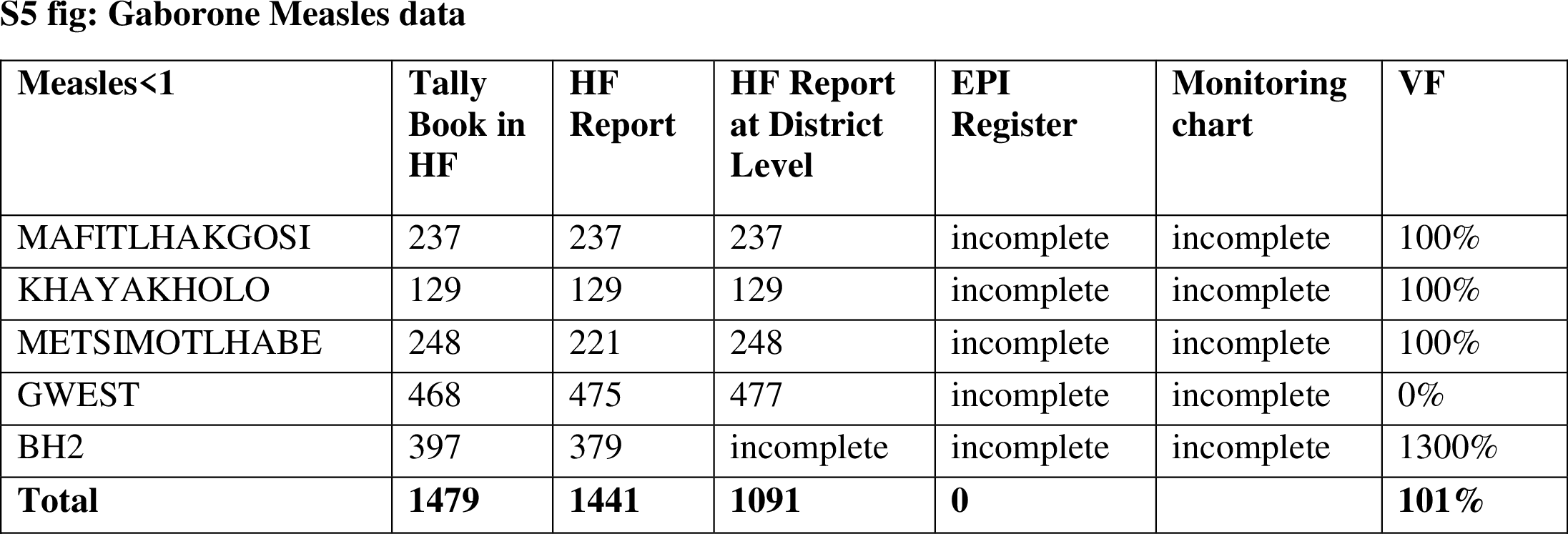

Supplement: S5 Fig — (TIF) [file pone.0220313.s005.tif]

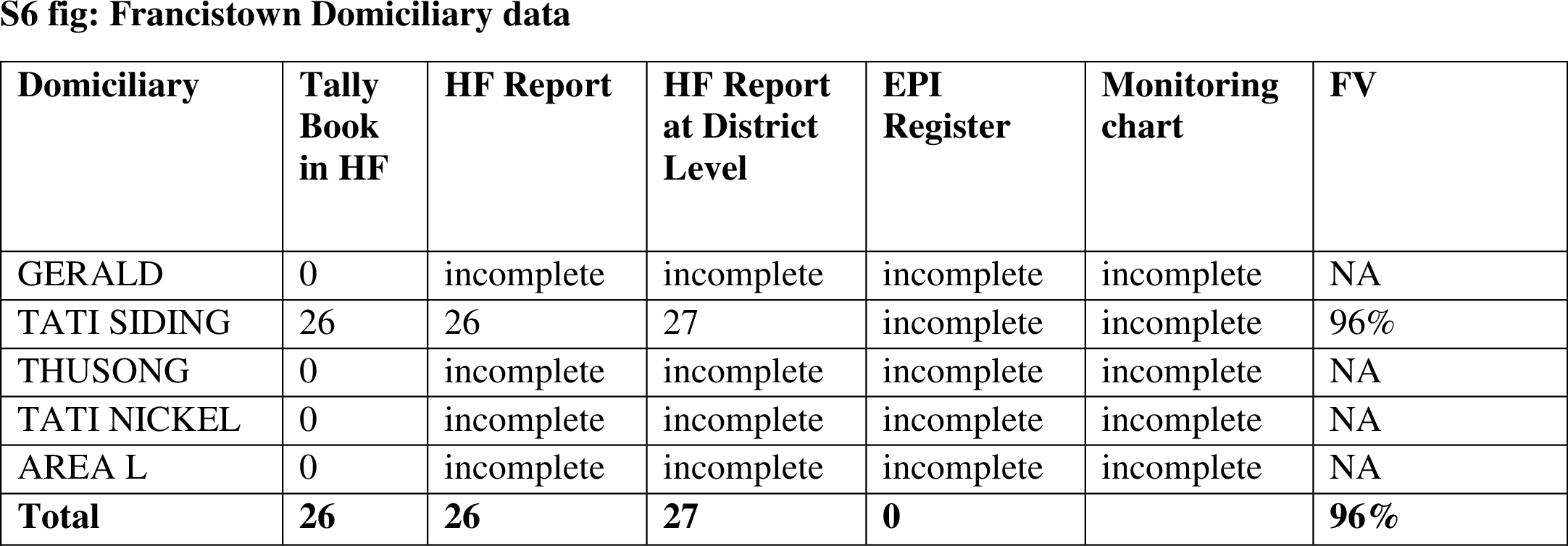

Supplement: S6 Fig — (TIF) [file pone.0220313.s006.tif]

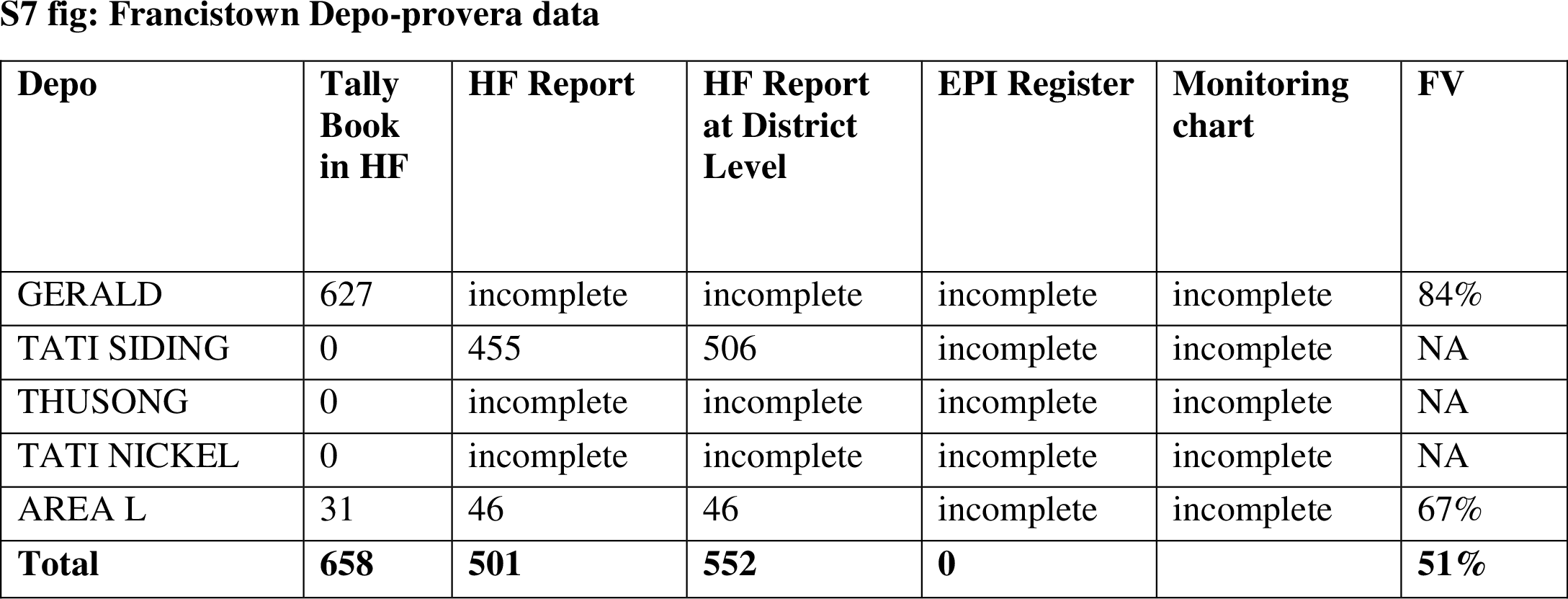

Supplement: S7 Fig — (TIF) [file pone.0220313.s007.tif]

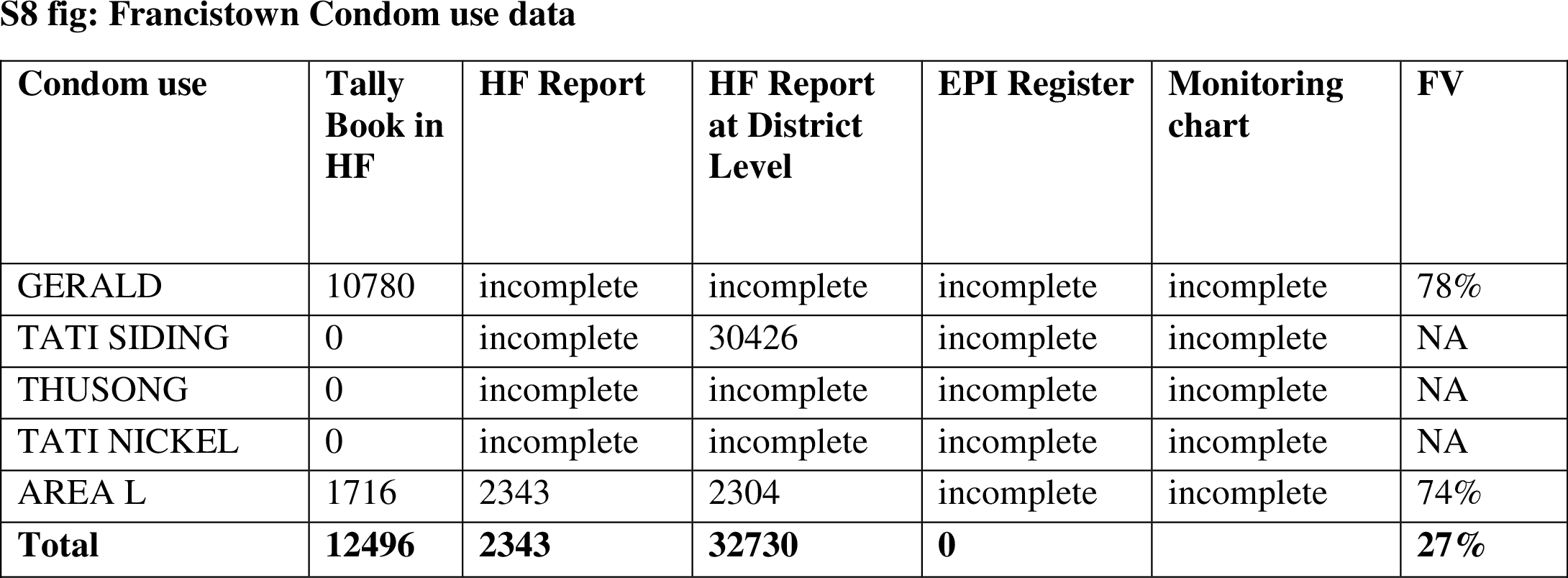

Supplement: S8 Fig — (TIF) [file pone.0220313.s008.tif]

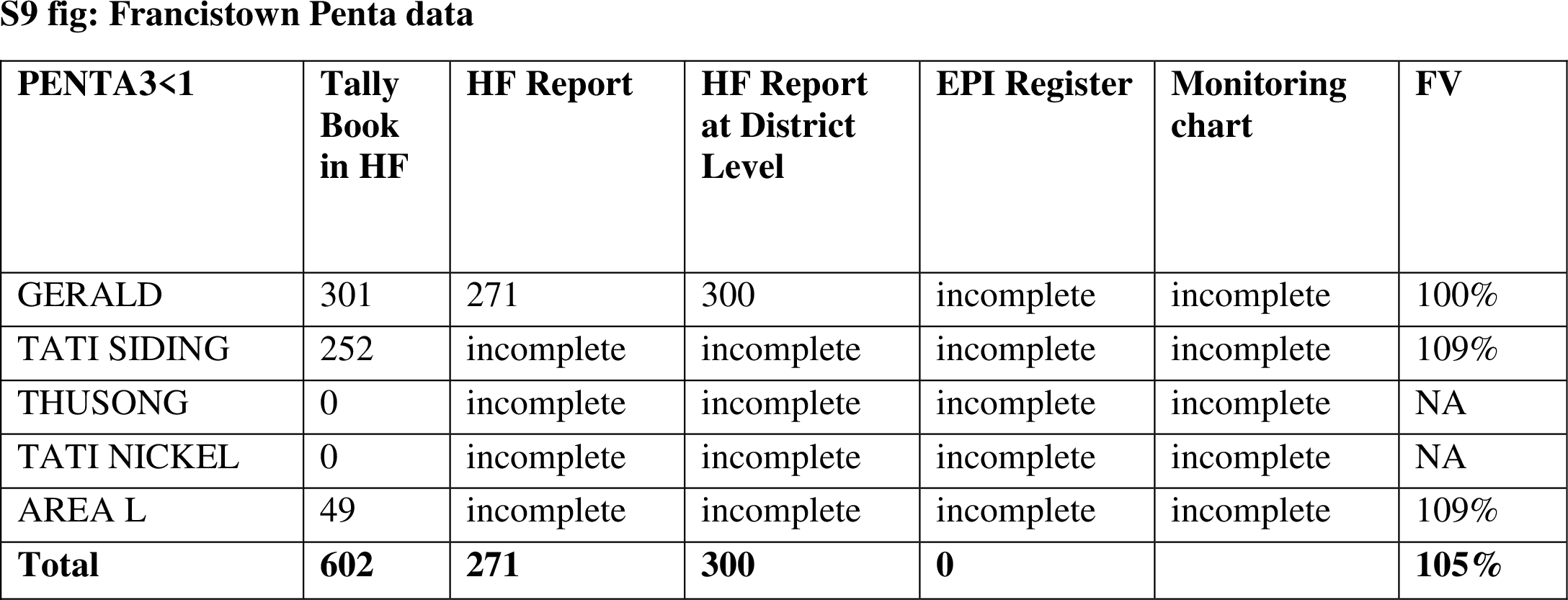

Supplement: S9 Fig — (TIF) [file pone.0220313.s009.tif]

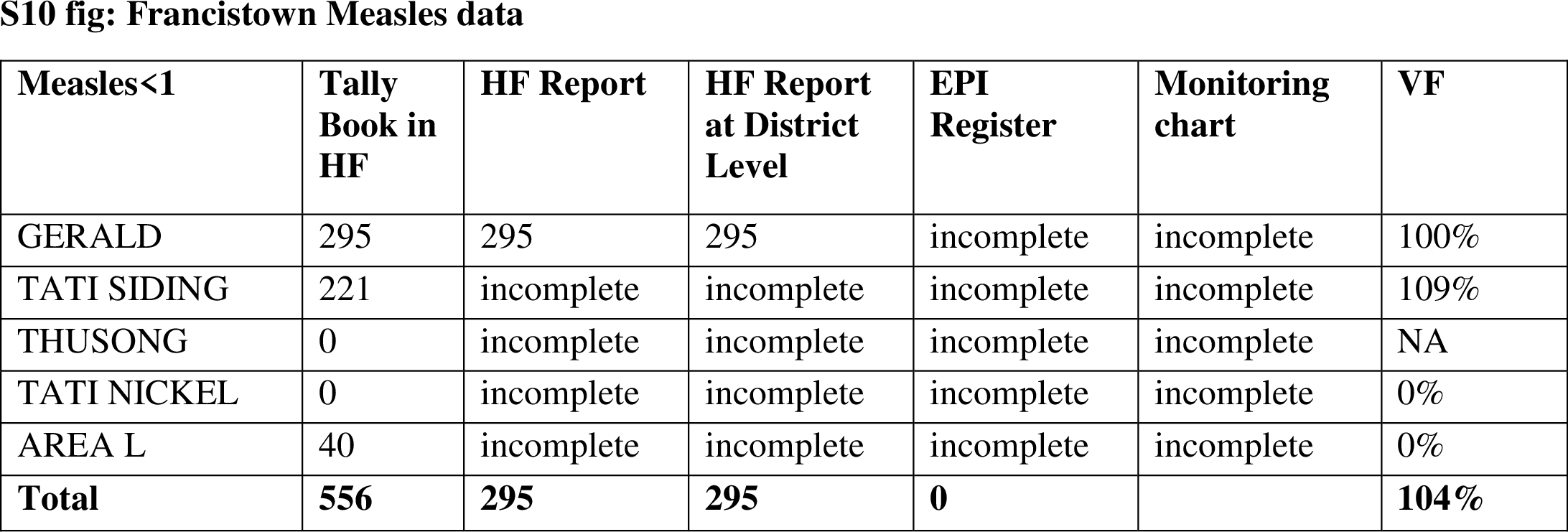

Supplement: S10 Fig — (TIF) [file pone.0220313.s010.tif]

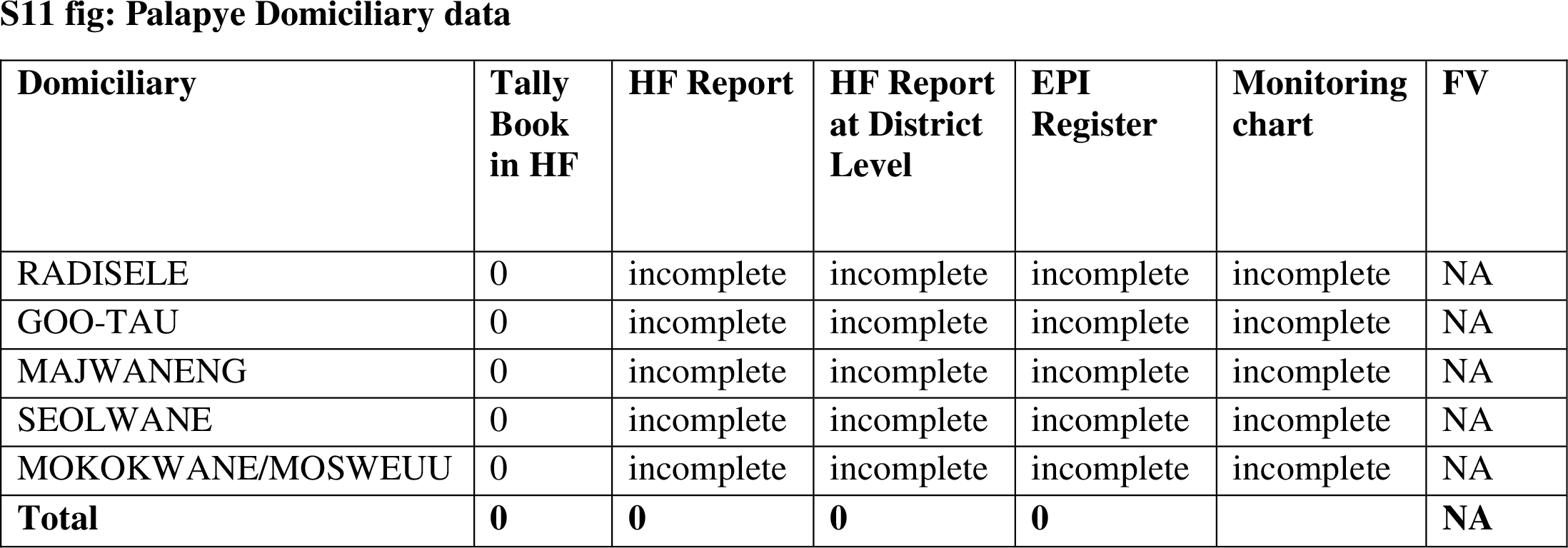

Supplement: S11 Fig — (TIF) [file pone.0220313.s011.tif]

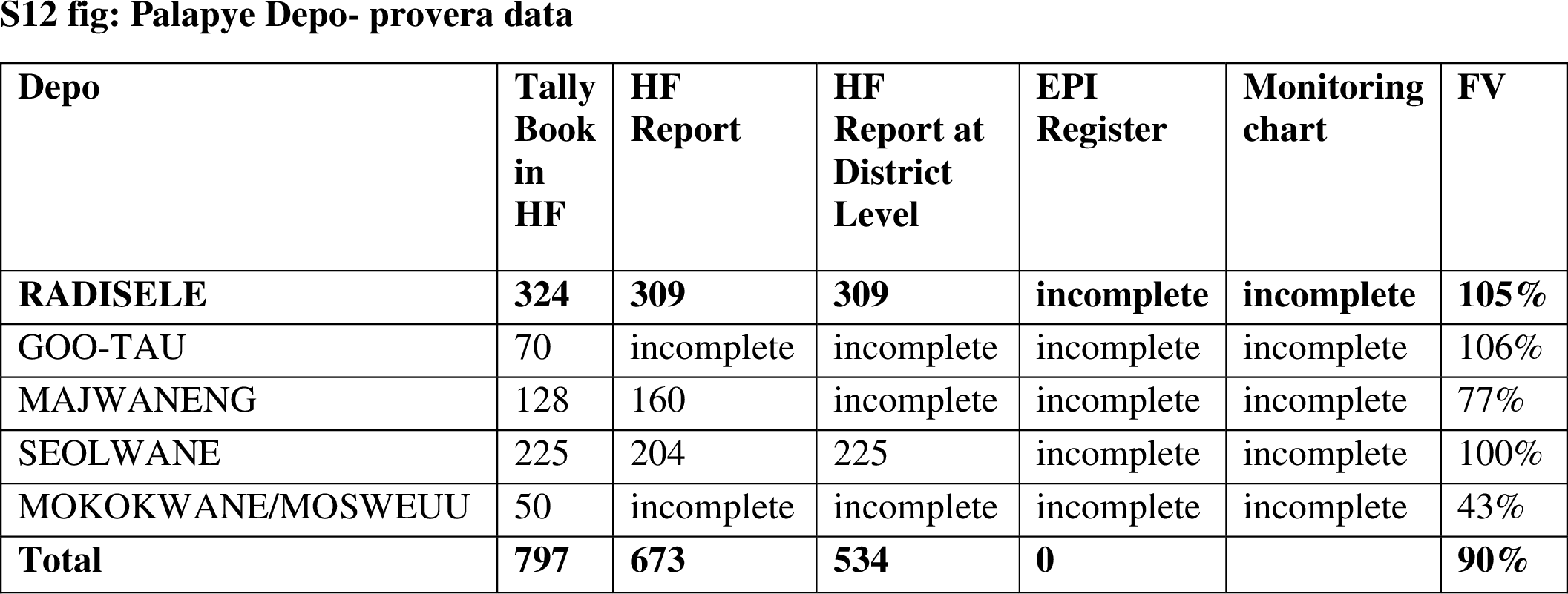

Supplement: S12 Fig — (TIF) [file pone.0220313.s012.tif]

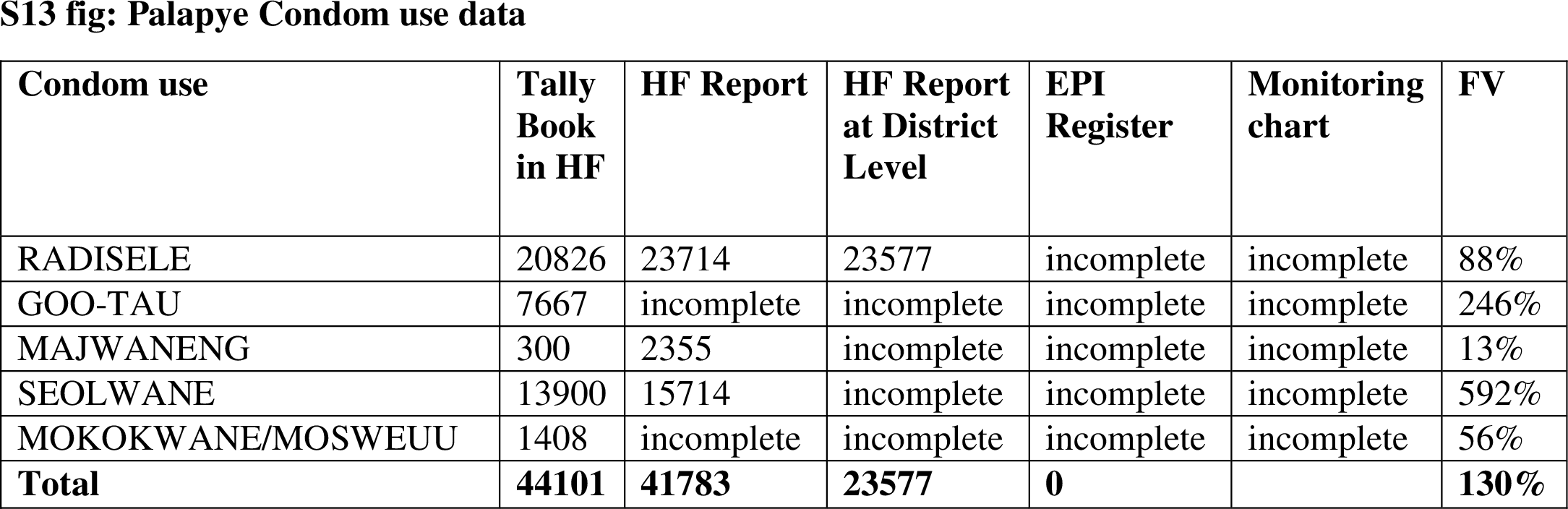

Supplement: S13 Fig — (TIF) [file pone.0220313.s013.tif]

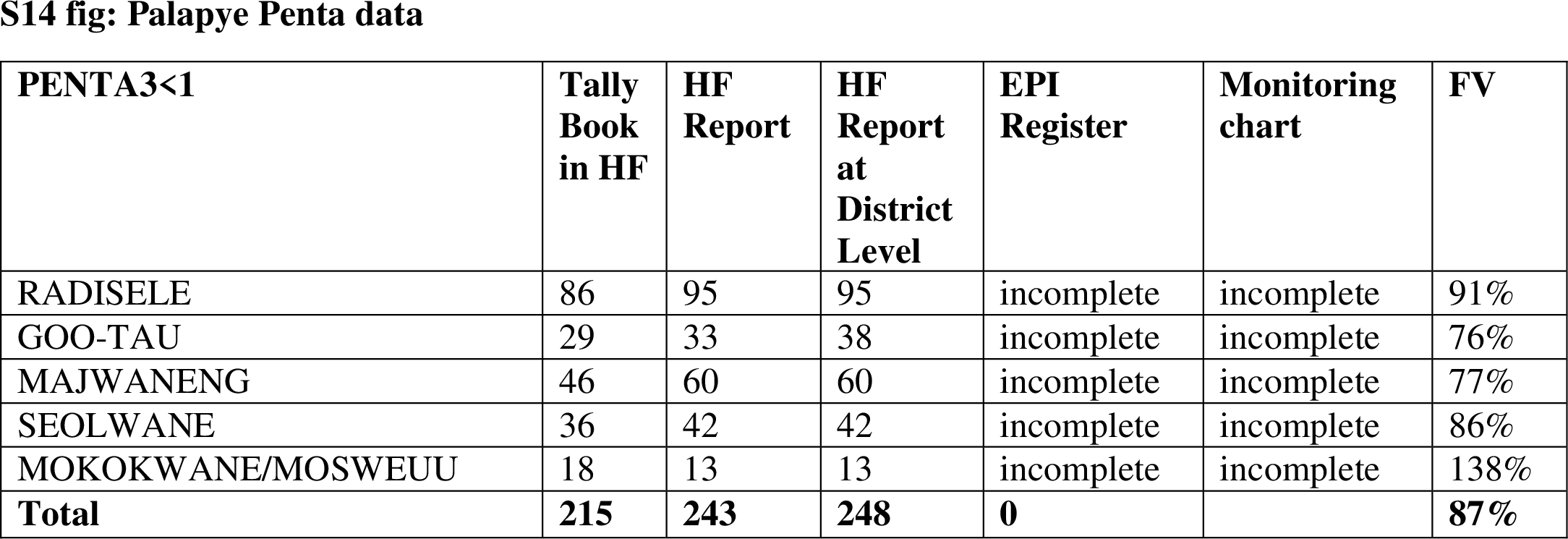

Supplement: S14 Fig — (TIF) [file pone.0220313.s014.tif]

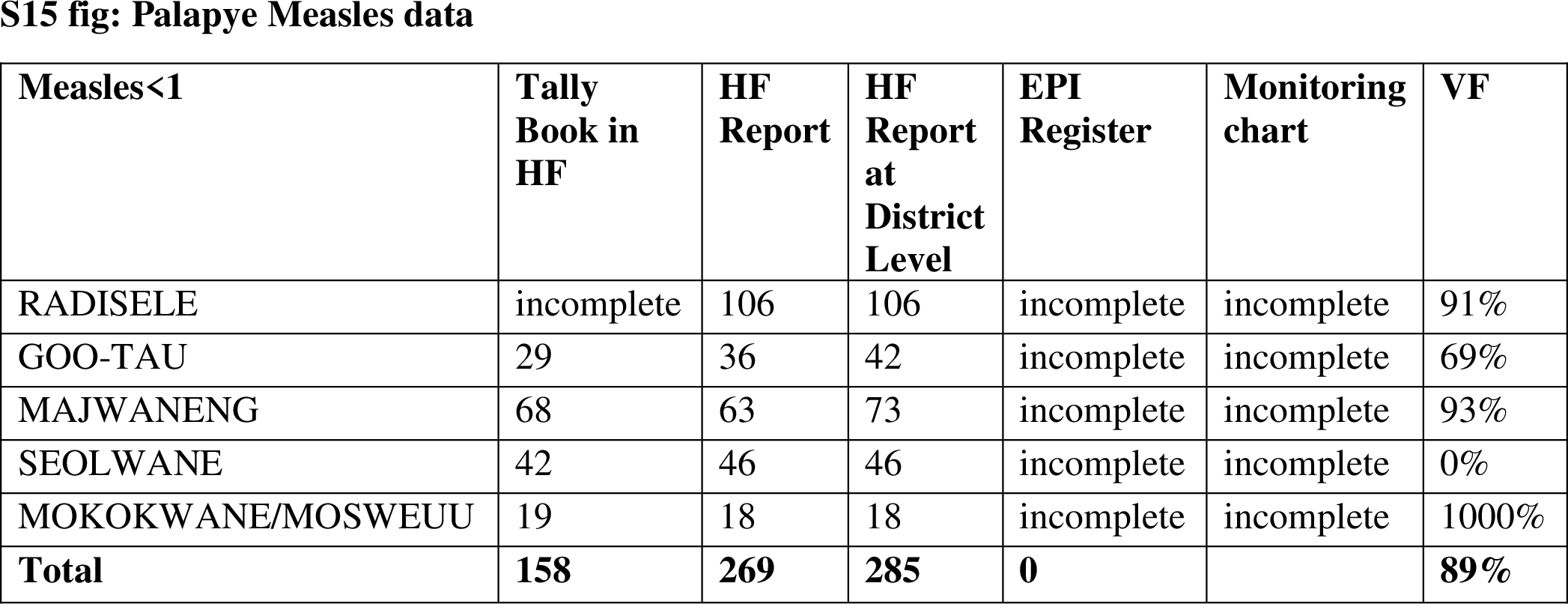

Supplement: S15 Fig — (TIF) [file pone.0220313.s015.tif]

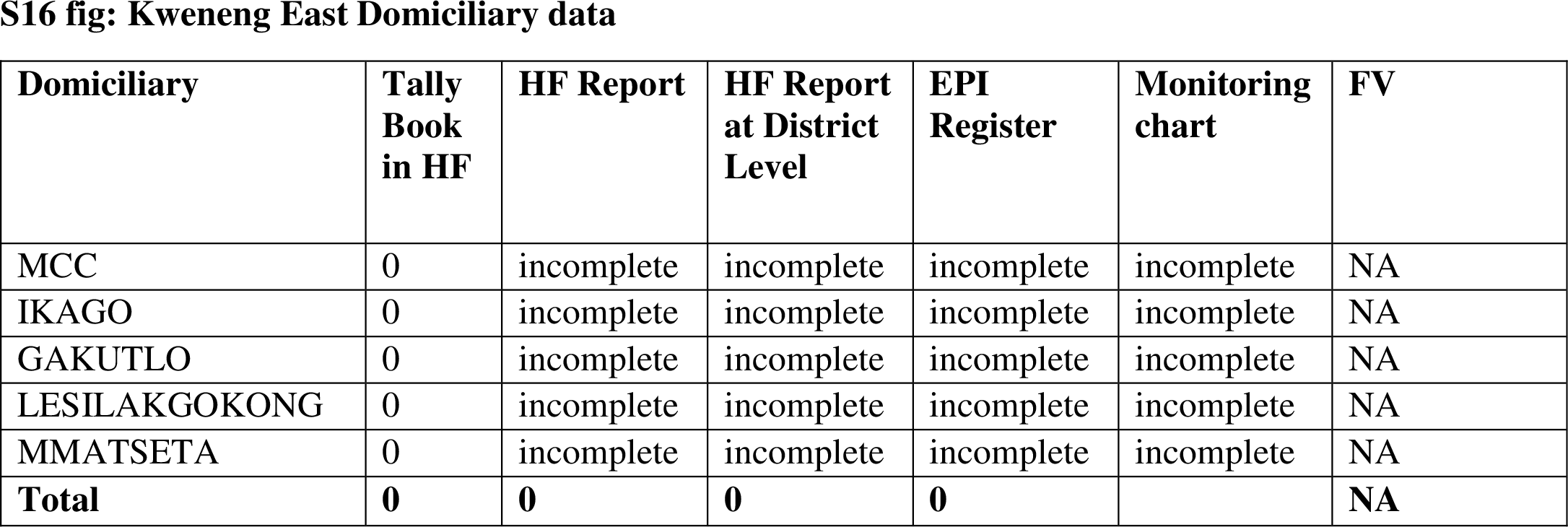

Supplement: S16 Fig — (TIF) [file pone.0220313.s016.tif]

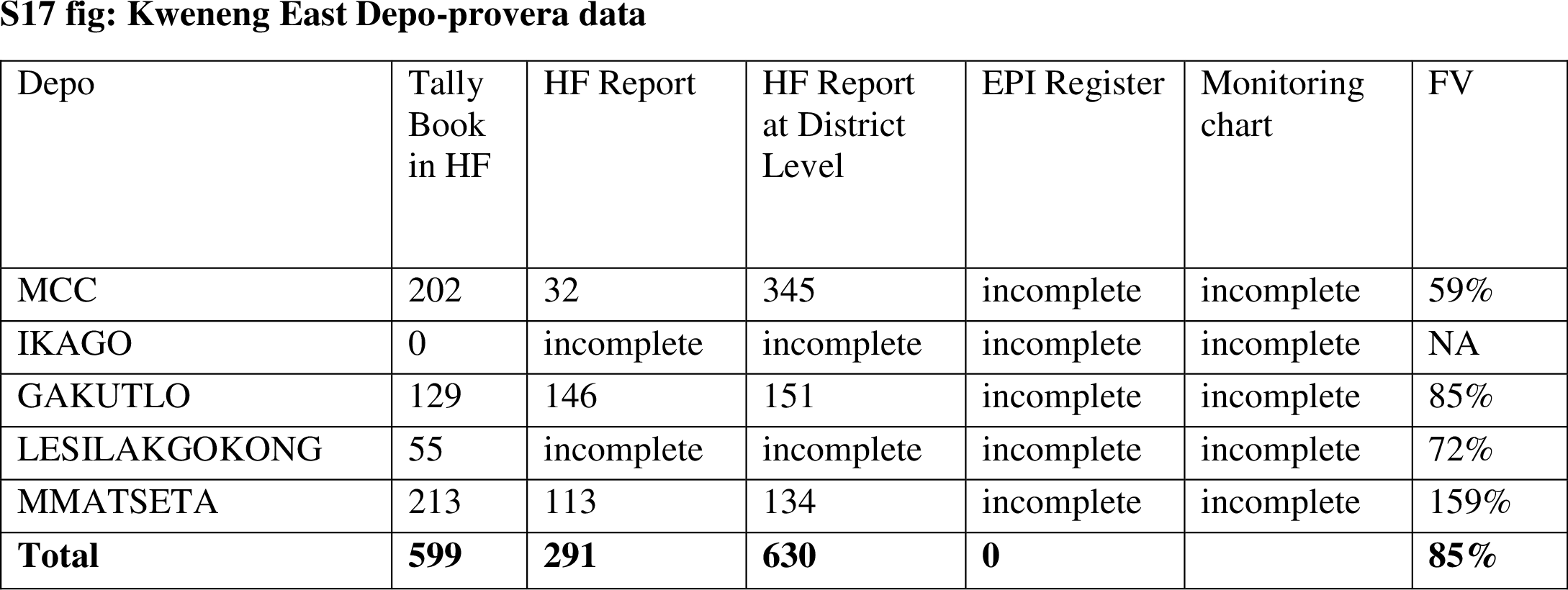

Supplement: S17 Fig — (TIF) [file pone.0220313.s017.tif]

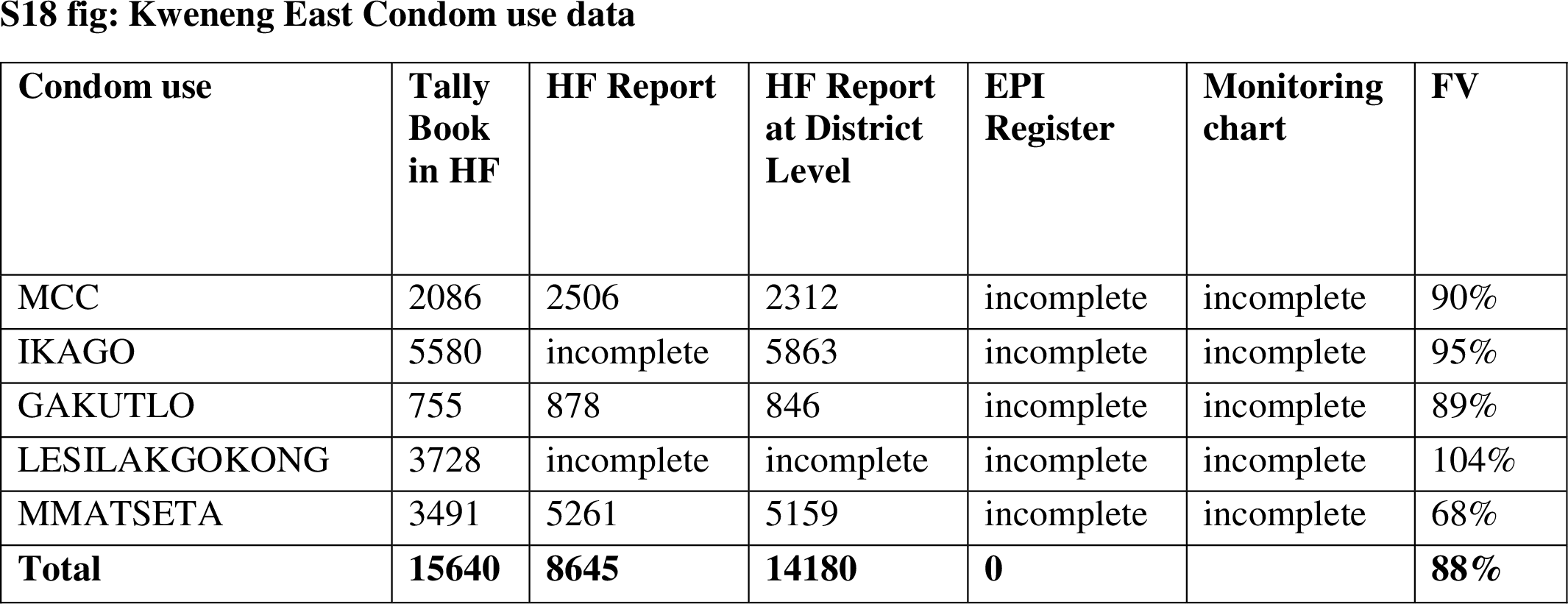

Supplement: S18 Fig — (TIF) [file pone.0220313.s018.tif]

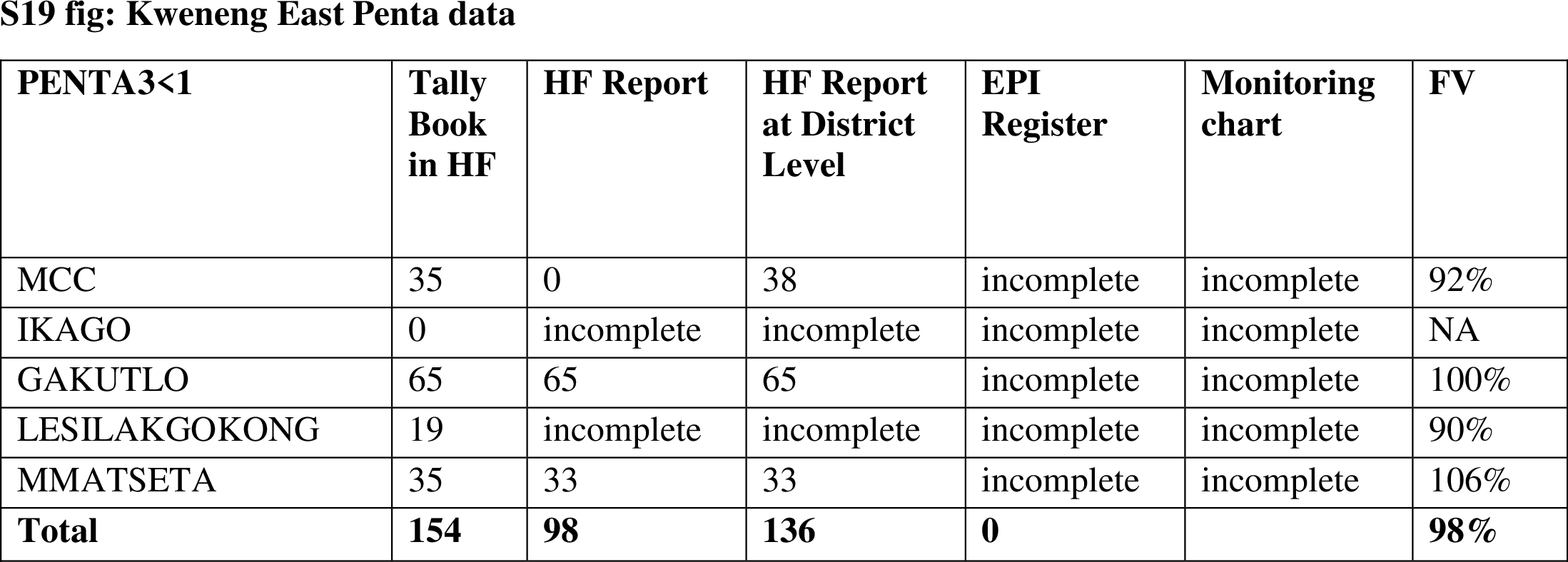

Supplement: S19 Fig — (TIF) [file pone.0220313.s019.tif]

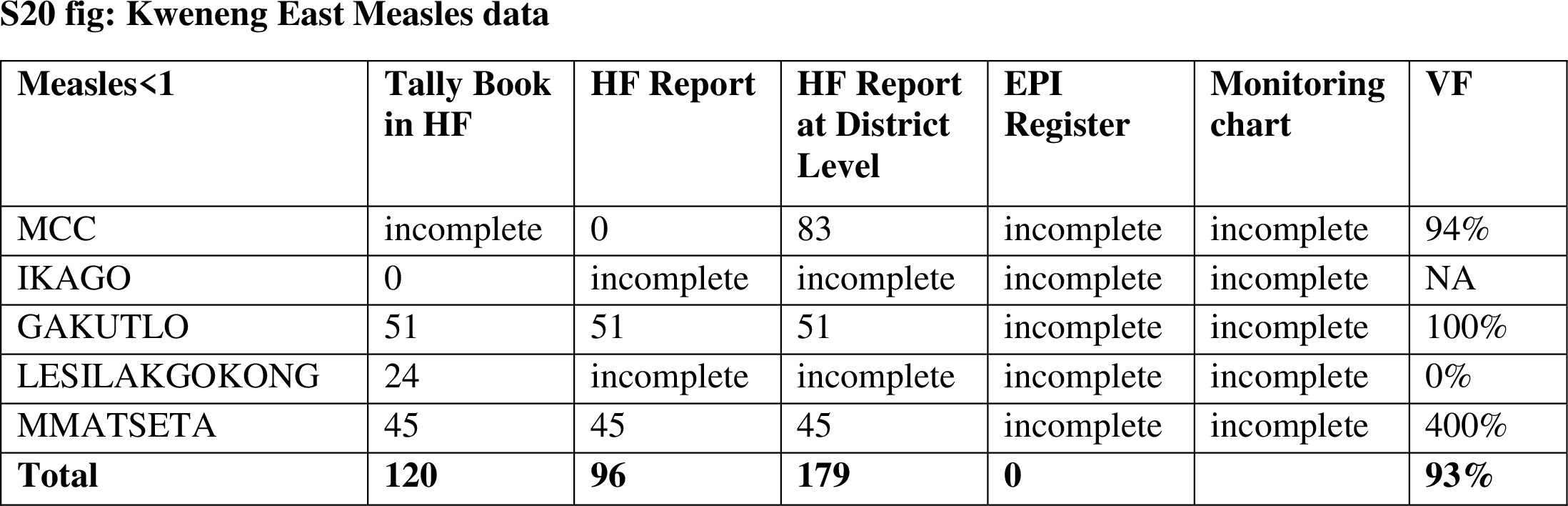

Supplement: S20 Fig — (TIF) [file pone.0220313.s020.tif]

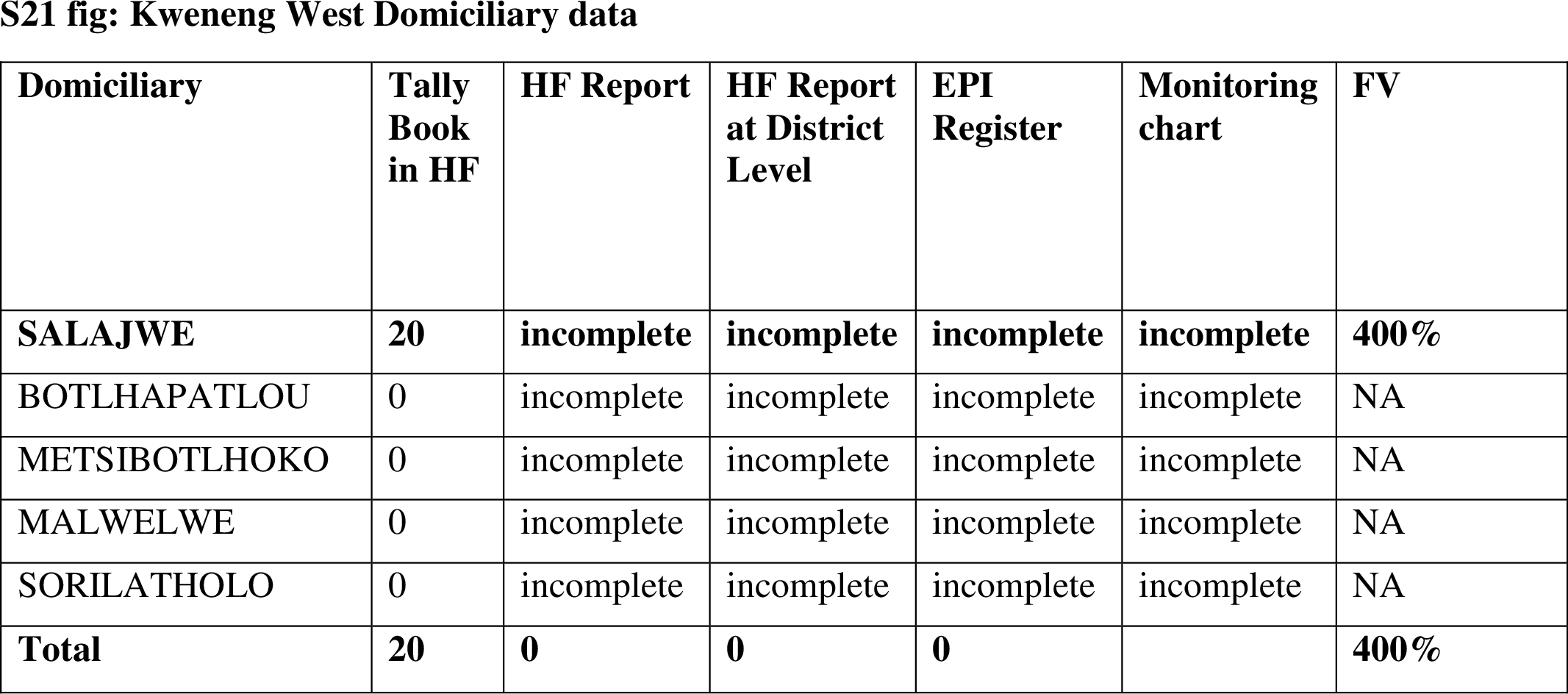

Supplement: S21 Fig — (TIF) [file pone.0220313.s021.tif]

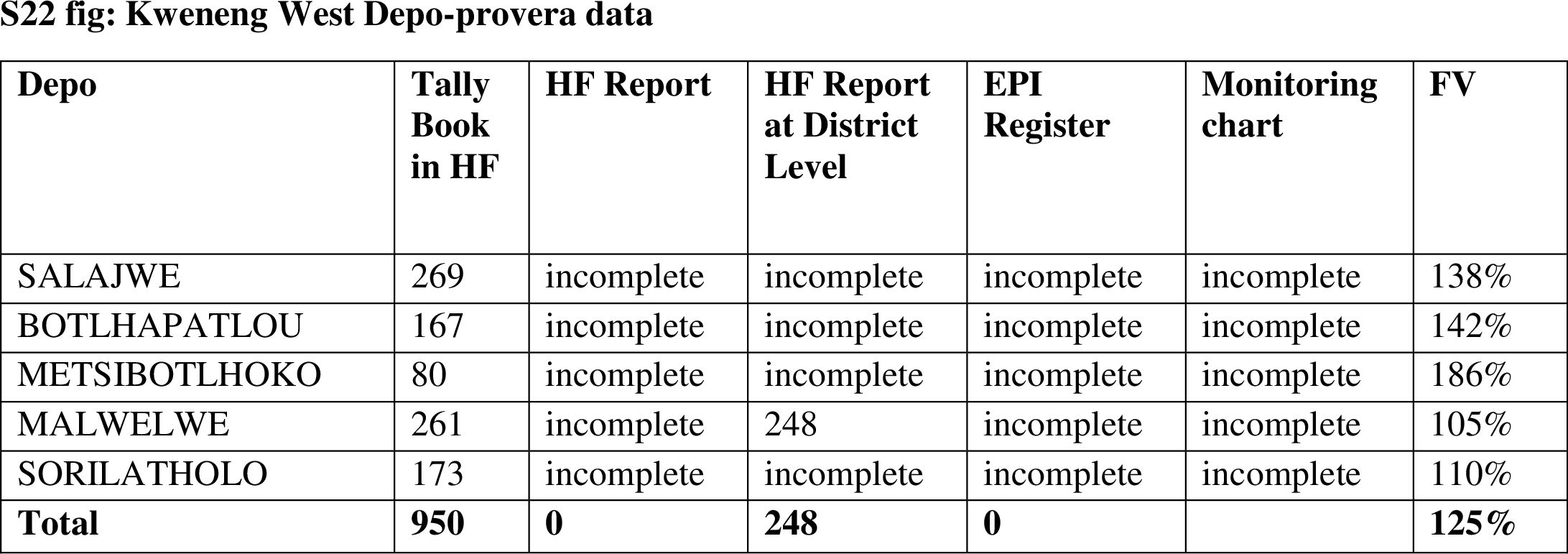

Supplement: S22 Fig — (TIF) [file pone.0220313.s022.tif]

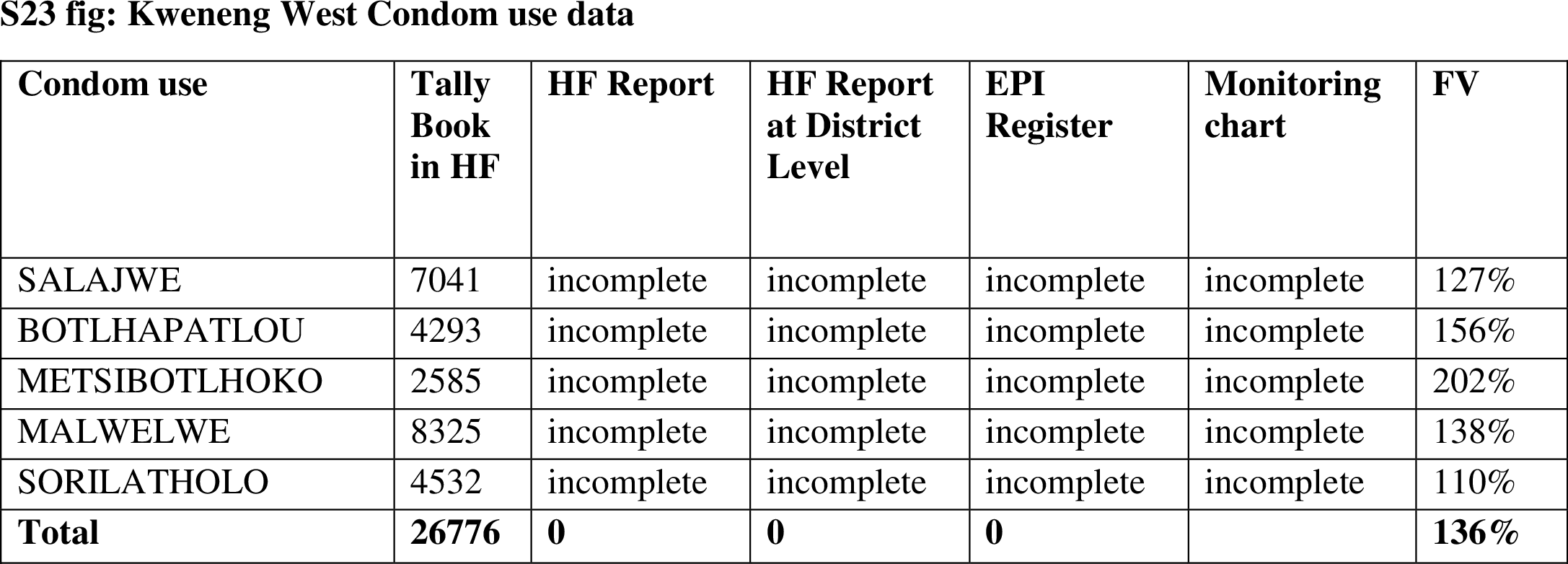

Supplement: S23 Fig — (TIF) [file pone.0220313.s023.tif]

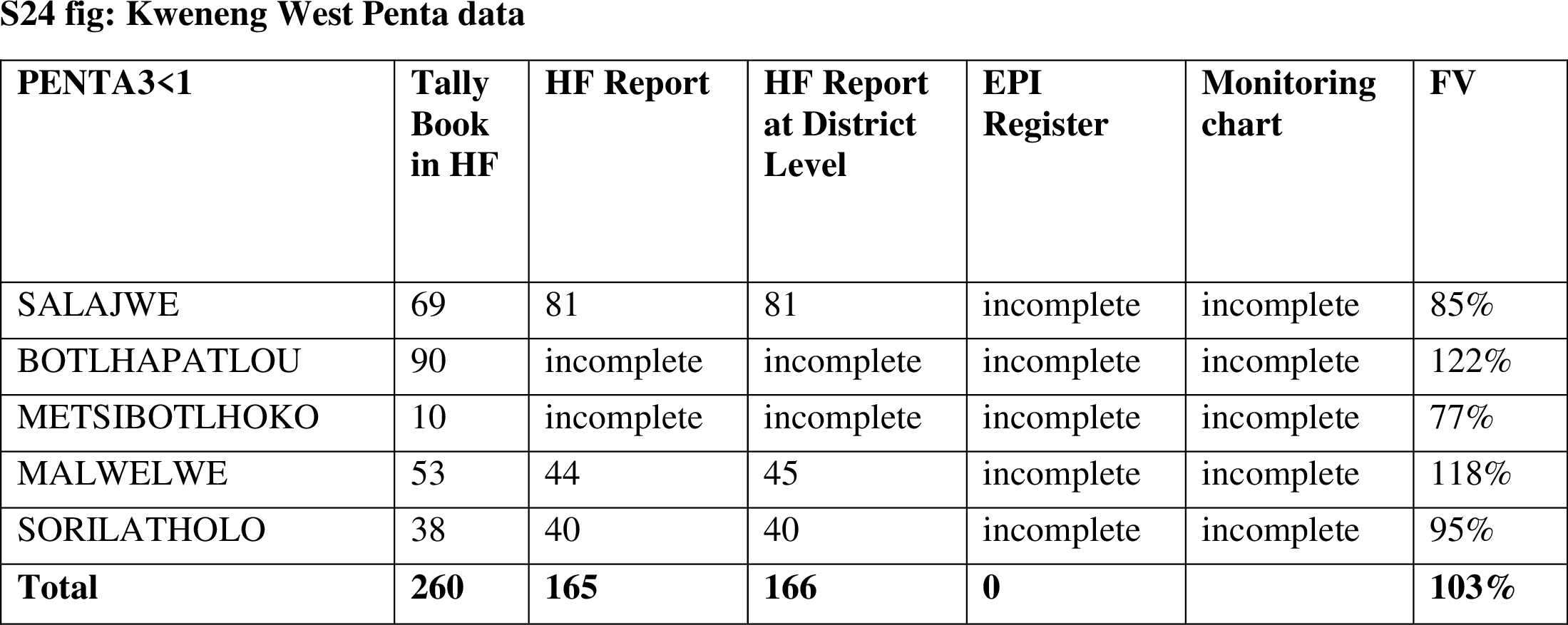

Supplement: S24 Fig — (TIF) [file pone.0220313.s024.tif]

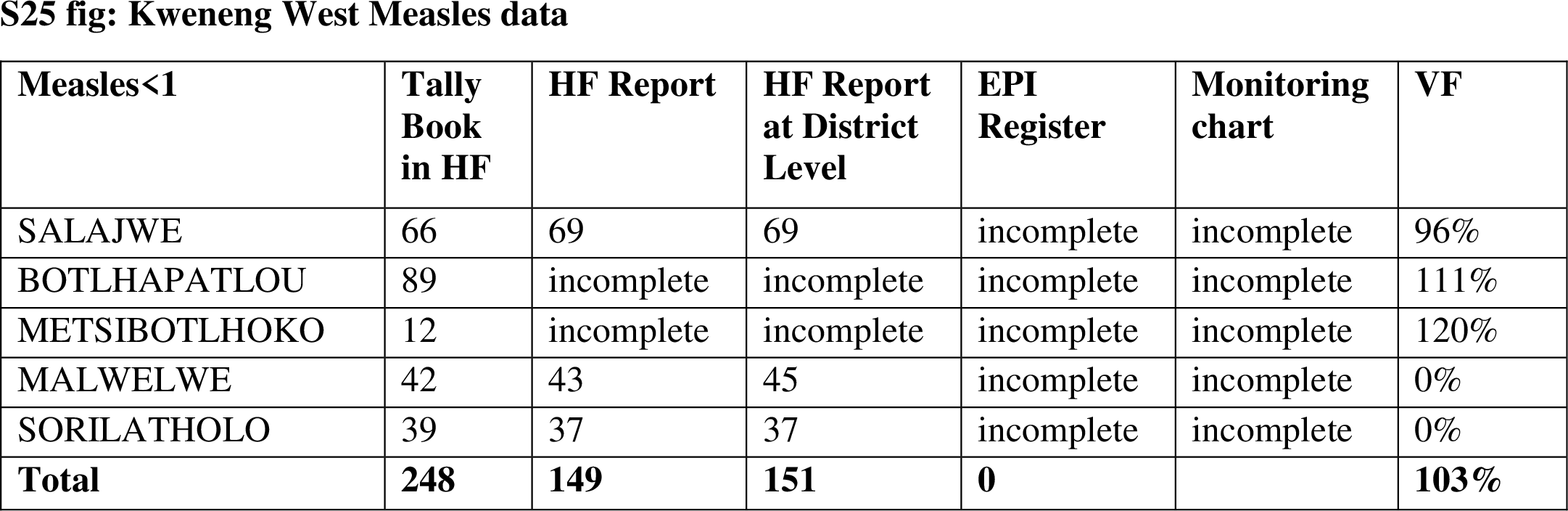

Supplement: S25 Fig — (TIF) [file pone.0220313.s025.tif]

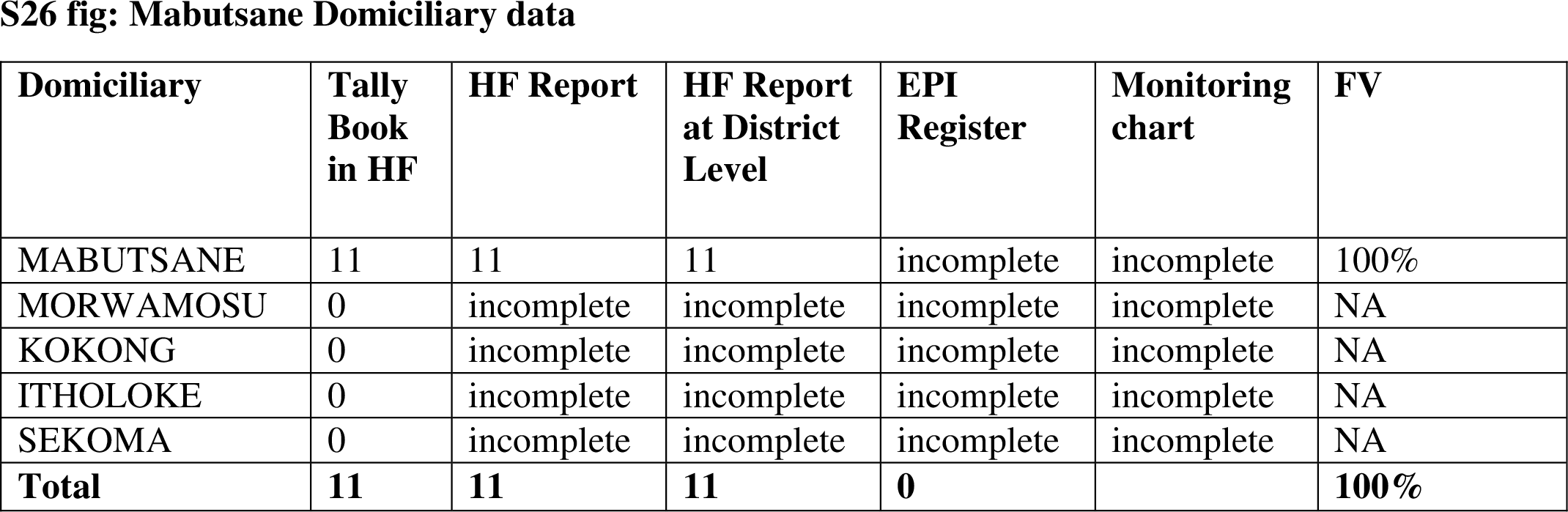

Supplement: S26 Fig — (TIF) [file pone.0220313.s026.tif]

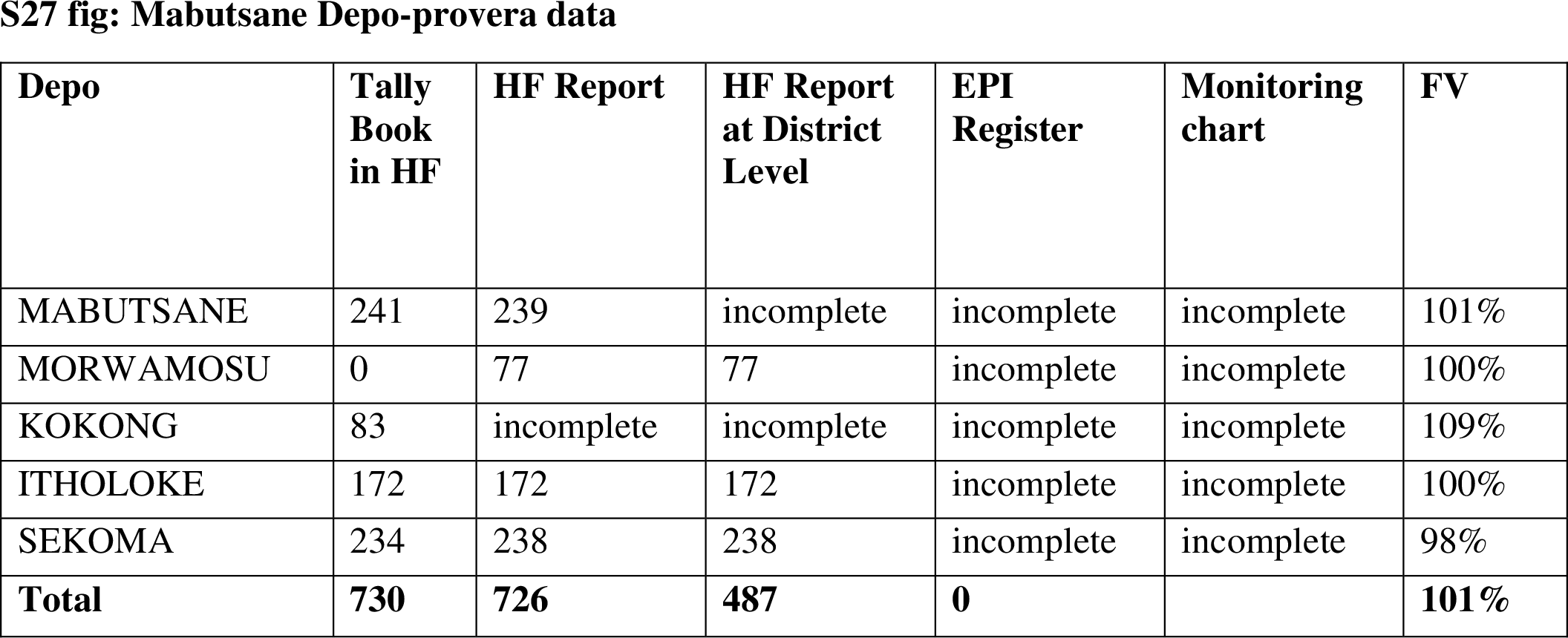

Supplement: S27 Fig — (TIF) [file pone.0220313.s027.tif]

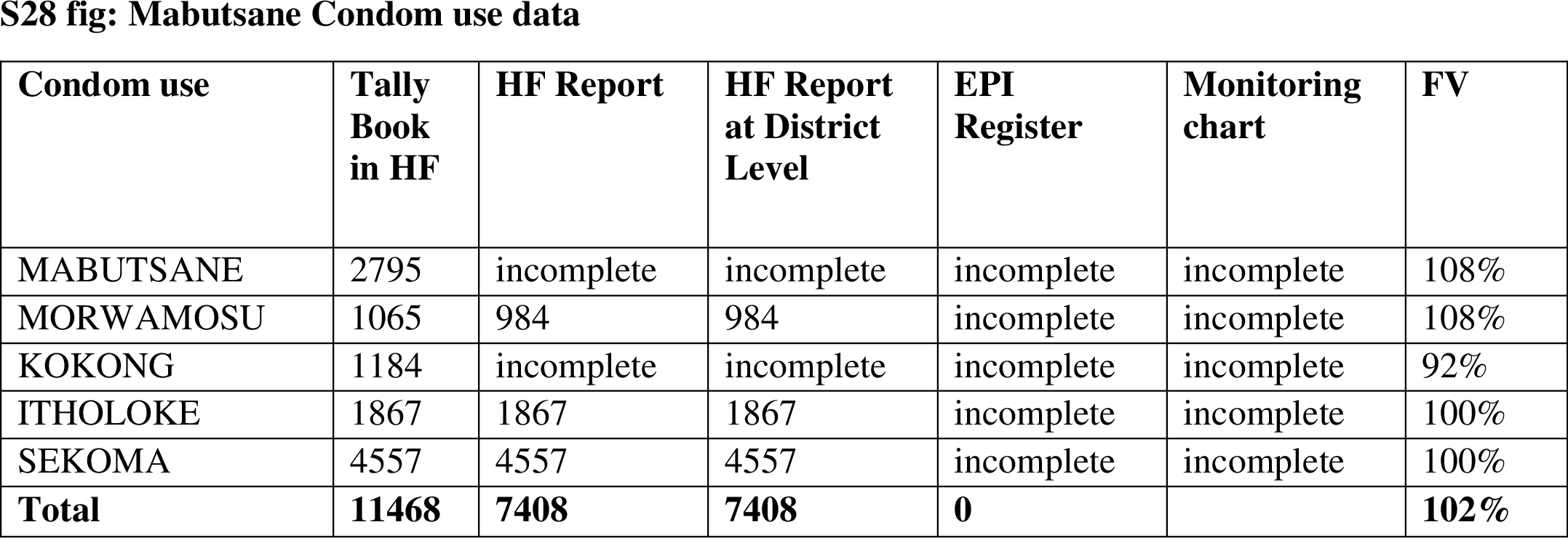

Supplement: S28 Fig — (TIF) [file pone.0220313.s028.tif]

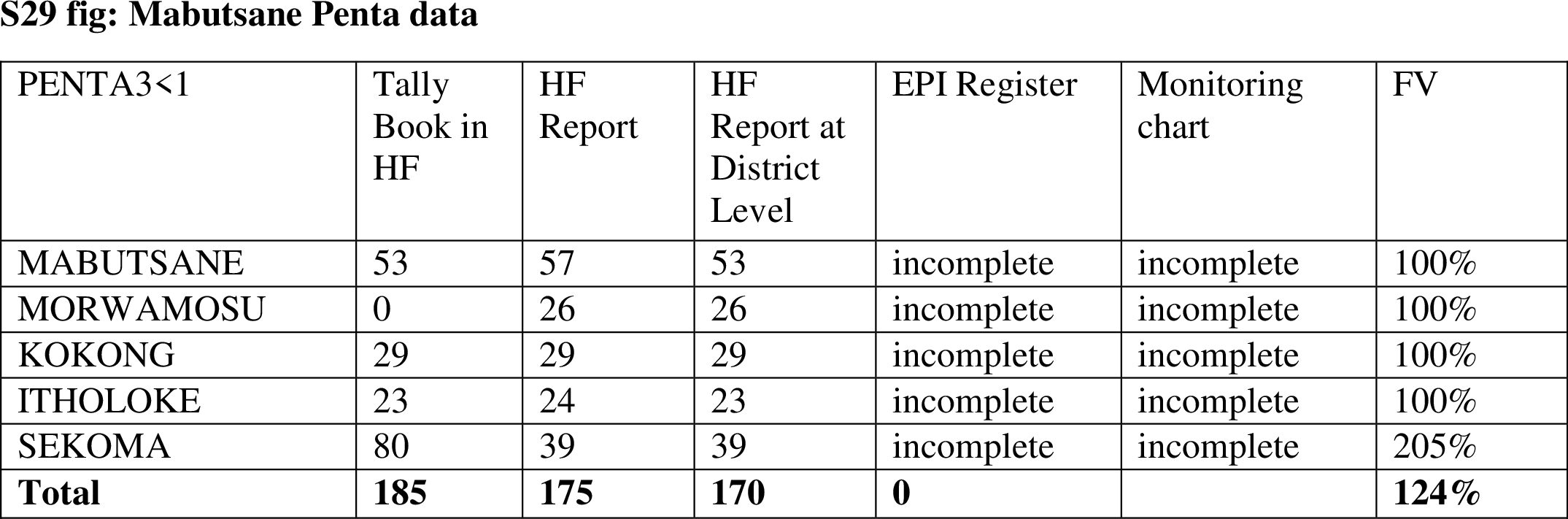

Supplement: S29 Fig — (TIF) [file pone.0220313.s029.tif]

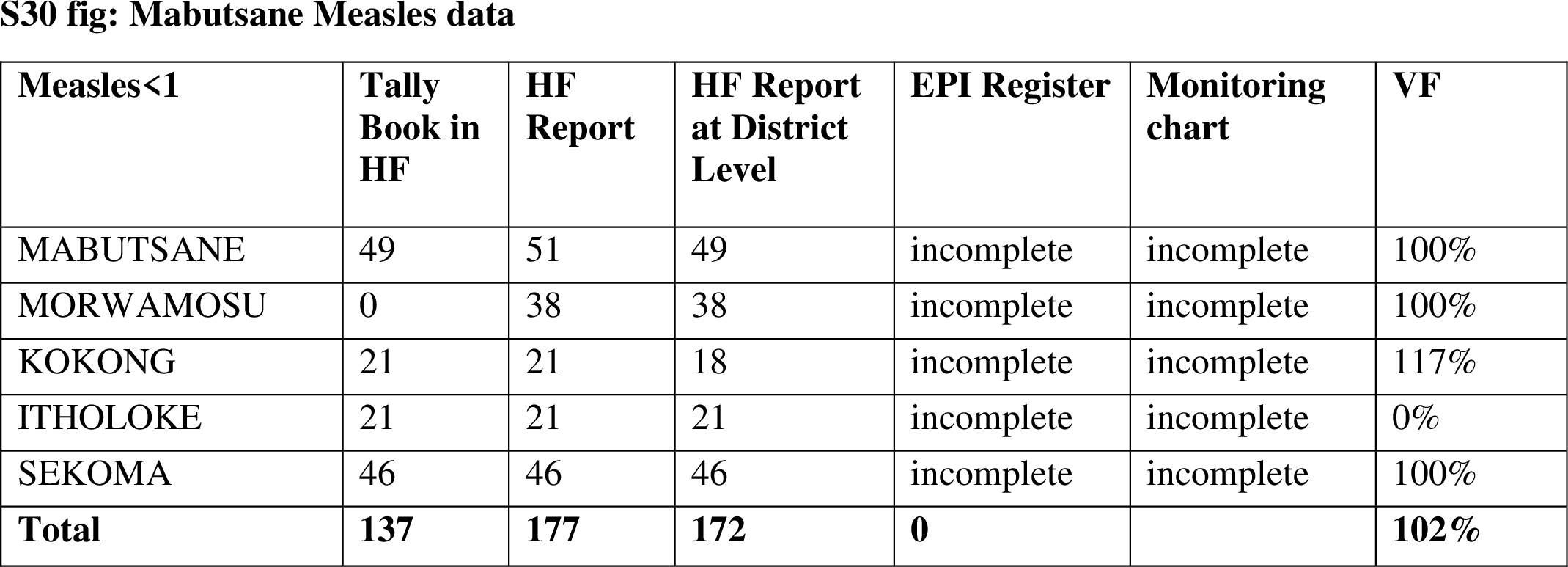

Supplement: S30 Fig — (TIF) [file pone.0220313.s030.tif]
